# Multi-dimensional single-cell characterization revealed suppressive immune microenvironment in AFP-positive hepatocellular carcinoma

**DOI:** 10.1038/s41421-023-00563-x

**Published:** 2023-06-19

**Authors:** Huisi He, Shuzhen Chen, Zhecai Fan, Yaping Dong, Ying Wang, Shiyao Li, Xiaojuan Sun, Yuting Song, Jinxian Yang, Qiqi Cao, Jie Jiang, Xianming Wang, Wen Wen, Hongyang Wang

**Affiliations:** 1 Third Affiliated Hospital of Naval Medical University, National Center for Liver Cancer, Shanghai, China; 2 International Cooperation Laboratory on Signal Transduction, Third Affiliated Hospital of Naval Medical University (Second Military Medical University), Shanghai, China; 3 Fudan University Shanghai Cancer Center, Department of Oncology, Shanghai Medical College, Fudan University, Shanghai, China; 4 Department of Laboratory Diagnosis, Third Affiliated Hospital of Naval Medical University (Second Military Medical University), Shanghai, China

**Keywords:** Liver cancer, Cancer microenvironment

## Abstract

Alpha-fetoprotein (AFP)-secreting hepatocellular carcinoma (HCC), which accounts for ~75% of HCCs, is more aggressive with a worse prognosis than those without AFP production. The mechanism through which the interaction between tumors and the microenvironment leads to distinct phenotypes is not yet clear. Therefore, our study aims to identify the characteristic features and potential treatment targets of AFP-negative HCC (ANHC) and AFP-positive HCC (APHC). We utilized single-cell RNA sequencing to analyze 6 ANHC, 6 APHC, and 4 adjacent normal tissues. Integrated multi-omics analysis together with survival analysis were also performed. Further validation was conducted via cytometry time-of-flight on 30 HCCs and multiplex immunohistochemistry on additional 59 HCCs. Our data showed that the genes related to antigen processing and interferon-γ response were abundant in tumor cells of APHC. Meanwhile, APHC was associated with multifaceted immune distortion, including exhaustion of diverse T cell subpopulations, and the accumulation of tumor-associated macrophages (TAMs). Notably, TAM-SPP1^+^ was highly enriched in APHC, as was its receptor CD44 on T cells and tumor cells. Targeting the Spp1-Cd44 axis restored T cell function in vitro and significantly reduced tumor burden when treated with either anti-Spp1 or anti-Cd44 antibody alone or in combination with anti-Pd-1 antibody in the mouse model. Furthermore, elevated IL6 and TGF-β1 signaling contributed to the enrichment of TAM-SPP1^+^ in APHC. In conclusion, this study uncovered a highly suppressive microenvironment in APHC and highlighted the role of TAM-SPP1^+^ in regulating the immune microenvironment, thereby revealing the SPP1-CD44 axis as a promising target for achieving a more favorable immune response in APHC treatment.

## Introduction

Liver cancer is the fourth-leading cause of cancer-related death worldwide, with hepatocellular carcinoma (HCC) accounting for the majority of primary liver cancer. The estimated 5-year survival rate is ~12% in China^1,2^. Serum AFP at a cutoff of 400 ng/mL is by far the most widely used biomarker for HCC screening and diagnosis^3–5^. Previous studies showed that HCC patients with negative AFP ( < 20 ng/mL) in serum are usually consistent with a relatively better prognosis. Compared with AFP-negative HCC (ANHC, AFP < 20 ng/mL) patients, AFP-positive HCC (APHC, AFP ≥ 20 ng/mL) patients were more likely to feature a larger tumor diameter, more vessel invasion, more advanced BCLC, and TNM stage^6,7^.

The oncogenic effect of AFP has been widely debated. A previous study has observed increased proliferation of HCC cell lines in vitro following the administration of AFP^8^. Cytoplasmic AFP could promote the degradation of PTEN and activate the mTOR/AKT signaling^9^. Other than its role in regulating tumor cell function, the AFP-high HCC samples exhibited altered immunity-related pathways^10^. AFP produced by HCC shows a suppressive effect on natural killer (NK) cells and T cells in vitro. Dendritic cells (DCs) exposed to AFP showed an impaired effect in stimulating antigen-specific T cell activation and proliferation^11,12^. The role of AFP has been discussed in previous reports; however, the molecular features and immune landscape, which were closely relevant to the altered phenotypes of APHC and ANHC patients were seldom investigated.

The discrepancy of clinical and pathological features as well as distinct prognosis of APHC and ANHC patients demonstrated the heterogeneity of HCC. With the rapid understanding of tumor immune microenvironment (TIME) in these years, single-cell sequencing was considered an effective way to profile the cell types in TIME and give some clues to explain the heterogeneity between tumors^13–15^. In this study, we applied single-cell RNA sequencing (scRNA-seq) to clarify the heterogeneity of APHC and ANHC, which simultaneously profiled the transcriptome of malignant cells, immune cells, and stromal cells. We also systemically compared the microenvironment of tumors with matched non-malignant tissues from adjacent peritumoral tissues. Moreover, we recruited additional patients with intact clinical information to perform high-throughput immunohistochemistry (IHC), cytometry time-of-flight (CyTOF), and bulk RNA sequencing (RNA-seq) for validation. Understanding the heterogeneity of APHC and ANHC would lead to the identification of potential therapeutic targets benefiting HCC patients. Our findings revealed a suppressive immune microenvironment and more TAM-SPP1^+^ in APHC than ANHC, which appeared to be associated with T cell exhaustion and tumor cell evasion. The underlying mechanism involved in the abundance of TAM-SPP1^+^ was also explored.

## Results

### Cohort characterization and single-cell analysis of multicellular ecosystem between ANHC and APHC

**Fig. 1:**
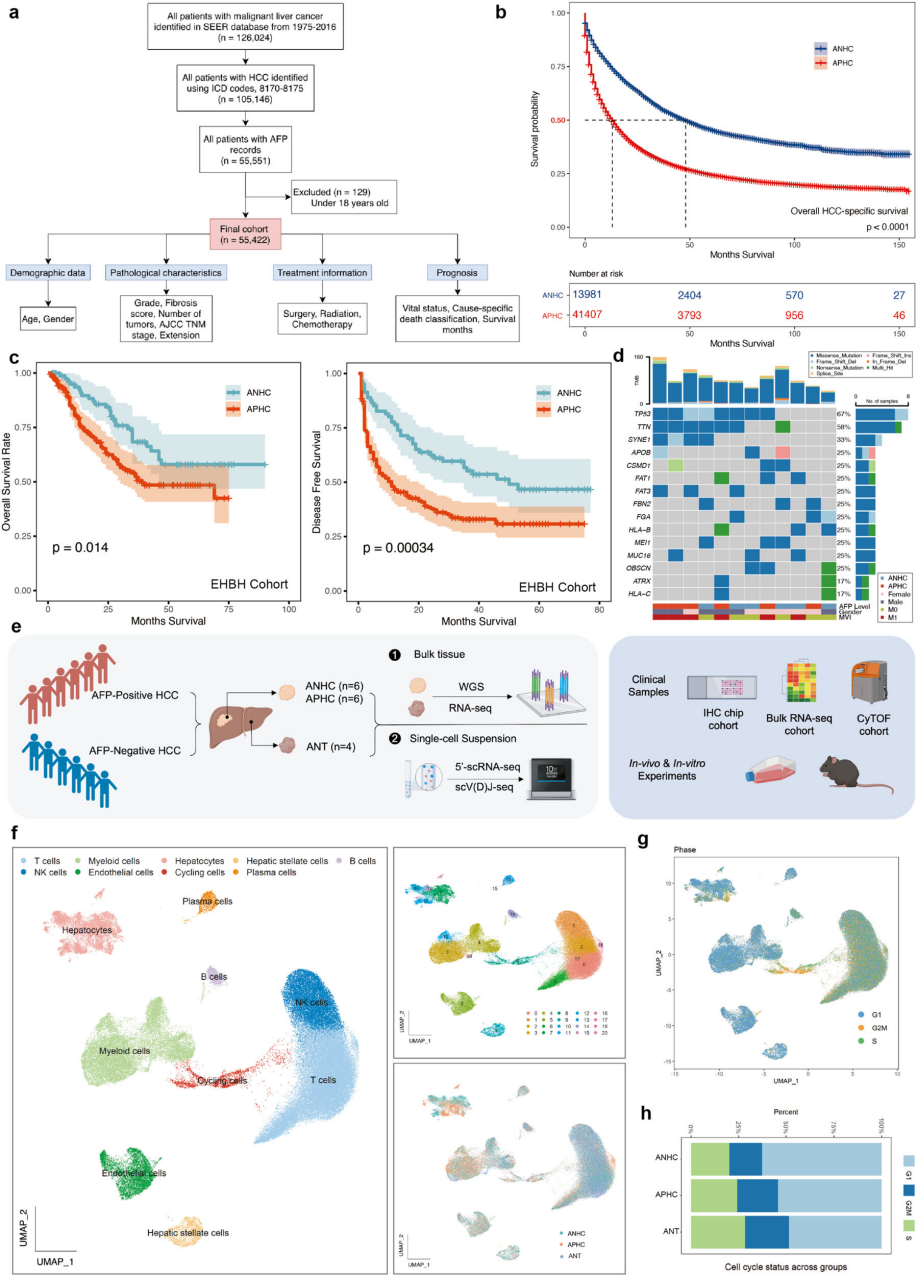
Cohort study and scRNA-seq profiling of ANHC and APHC patients. **a** Flow diagram of study cohort selection based on SEER database. **b** Overall survival curves of ANHC (*n* = 13,990) vs APHC (*n* = 41,432) patients from the SEER database (partial survival data has been lost within this database). **c** Kaplan-Meier plots of overall survival (left) and disease-free survival (right) in ANHC (*n* = 85) and APHC (*n* = 212) patients. **d** WGS analysis of the patients from the scRNA-seq cohort. Top histogram, TMB; right histogram, frequency of mutated genes (genes with mutations in > 2 samples are displayed); below tracks, clinical and pathologic characteristics. **e** Schematic representation of the study design. The discovery cohort is shown on the left and validation strategies were used (right). **f** Uniform Manifold Approximation and Projection (UMAP) plot depicts 92,762 cells representing 9 major cell lineages across three groups. **g**, **h** Cell cycle heterogeneity and corresponding proportions of cells within ANHC, APHC, and ANT.

To characterize the clinical and pathological features of ANHC (serum AFP < 20 ng/mL at diagnosis) and APHC (serum AFP level ≥ 20 ng/mL at diagnosis), two patient cohorts were analyzed. One retrospective study utilized data from the Surveillance, Epidemiology, and End Results (SEER) database, providing information on cancer statistics^16,17^. The second cohort consisted of patients who underwent radical hepatectomy for primary HCC at the Eastern Hepatobiliary Hospital (EHBH). Within the SEER cohort, 55,422 eligible patients with traceable clinical records were enrolled, including 13,990 ANHC and 41,432 APHC, which indicated that about a quarter of patients were AFP negative (Fig. 1a). The clinical and pathological characteristics of this cohort were presented in Supplementary Table S1. Consistent with previous reports, APHC presented more aggressive biological behavior with higher fibrosis scores, TNM classification, clinical stage, and a more advanced invasive pattern. The median overall survival (OS) in the APHC group was much shorter than that in the ANHC group (13 months vs 48 months, Fig. 1b) regardless of whether surgery or adjuvant therapy was performed (Supplementary Fig. S1a), indicating that patients with APHC showed a significantly worse prognosis.

Similarly, distribution differences in demographic characteristics and histological features were observed between the two groups in the EHBH cohort (*n* = 297, Supplementary Table S2), which further confirmed that APHC (*n* = 212, 71.38%) displayed more advanced progression than ANHC (*n* = 85, 28.62%). As shown in Fig. 1c, patients with elevated AFP levels had poor OS and disease-free survival (DFS) compared with patients with ANHC (APHC vs ANHC: OS, 39 months vs not evaluable; DFS, 11 months vs 50 months). Therefore, the above data strongly suggest a more aggressive progression and worse prognosis in APHC, and the underlying mechanism needs to be investigated.

Firstly, whole-genome sequencing (WGS) data generated from matched tumors (ANHC, *n* = 6; APHC, *n* = 6) and adjacent normal tissue (ANT) were used to analyze genomic alterations typical for APHC and ANHC. In general, > 60% of tumors displayed *TP53* mutation and > 50% of patients had mutations in *TTN* encoding TITIN (Fig. 1d), both of which were more abundant in the APHC group. However, ANHC and APHC were not well characterized at the genome-wide level.

To construct a global tumor atlas of HCC with different serum AFP levels, the same samples used for WGS (ANHC, *n* = 6; APHC, *n* = 6) were subjected to scRNA-seq with 4 ANTs as control. Detailed clinical information of these 12 patients was provided in Supplementary Table S3. The validation cohorts were used subsequently to confirm the findings of the discovery cohort (Fig. 1e). After data pre-processing (Supplementary Fig. S1b), graph-based clustering analysis generated 92,762 high-quality single-cell transcriptomes for HCC and partitioned cells into 9 major clusters and 38 subsets in total based on canonical markers (Fig. 1f; Supplementary Fig. S1g). The distribution of major cell types in each group was presented (Supplementary Fig. S1c). Cell cycle state among all available cells across all three groups was identified and a larger proportion of cells in the G2/M state was observed in the APHC group than in ANHC or ANT group, indicating more active cell proliferation in APHC (Fig. 1g, h). The distribution of all cellular subsets of each sample was presented in Supplementary Fig. S1d. The composition of major cell types was highly variable between tumors and ANTs (Fig. 1f; Supplementary Fig. S1e), whereas the difference between the two tumor groups was not obvious within 9 major clusters (Supplementary Fig. S1f).

### The intrinsic immunogenic features of malignant cells in APHC

**Fig. 2:**
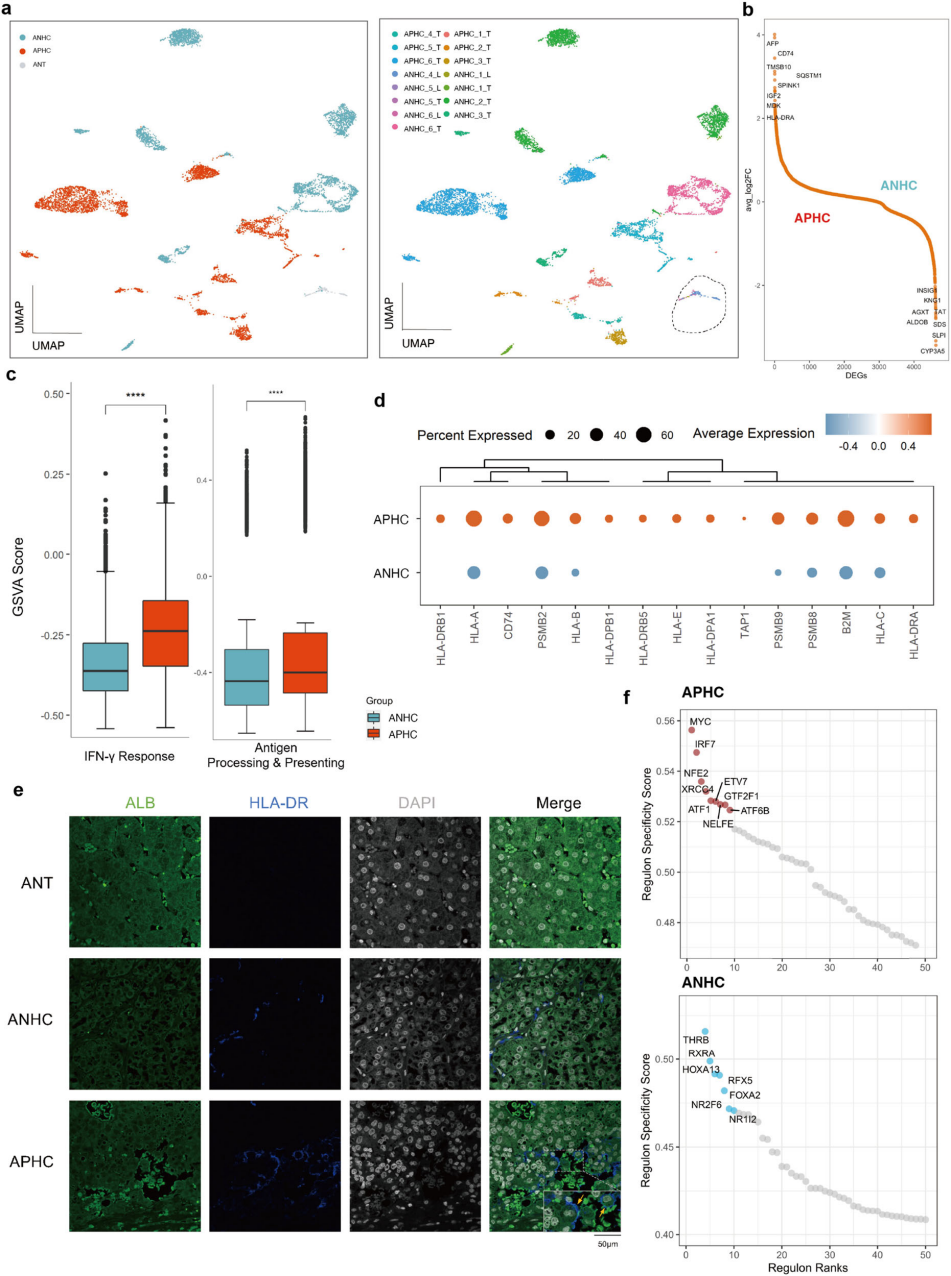
Deciphering the molecular features of malignant cells. **a** UMAP plot showing the patient-specific distribution feature of malignant cells. Black dotted circle, non-malignant cells. **b** Ranking of significantly altered genes in APHC compared with ANHC. The top 8 positive and negative differentially expressed genes (DEGs) are labeled. **c** Boxplots showing the IFN-γ response and antigen processing and presentation scores of malignant cells from ANHC and APHC. **d** Average expression of genes related to antigen presentation and processing in APHC and ANHC. **e** The multiplex IHC staining of HLA-DR in ANT, ANHC, and APHC (representative image from each group). **f** Scatter plot showing the specificity scores of regulons in malignant cells of ANHC and APHC.

The hepatocytes/tumor cells displayed chromosome-scale deviations of expressing magnitudes indicative of the presence of copy number variation (CNV) via the inferCNV algorithm. 8975 epithelial cells were identified in 16 samples, including 7864 malignant cells (ANHC, 3483 cells; APHC, 4481 cells) and 1011 nonmalignant cells (Supplementary Fig. S2a). We observed patient-specific clustering in the majority of cancer cell clusters, while nonmalignant cells in adjacent noncancerous tissue were clustered together (Fig. 2a).

We first investigated whether tumor-intrinsic features contributed to altered patterns of biological behaviors. By performing a differential analysis between the two tumor cell compartments, we found that numerous genes associated with antigen presentation (e.g., HLA-DRA, CD74) were intensively upregulated in APHC, while a variety of metabolic enzymes (e.g., CYP3A5, ALDOB) were highly expressed in ANHC (Fig. 2b). Tumor-specific major histocompatibility complex (MHC) class II (tsMHC-II) may increase tumor cell recognition by immune cells^18^ and predict the response to anti-PD-1/anti-PD-L1 therapy in melanoma and lung cancer^19,20^. Gene set variation analysis (GSVA) revealed that interferon-γ (IFN-γ) response, as well as antigen processing and presentation pathways, were significantly enriched in APHC (Fig. 2c). Notably, both MHC class I and MHC class II antigen presentation genes were highly expressed in APHC, especially for MHC class II (Fig. 2d). IHC and multiplex IHC validated elevated HLA-DR expression in tumor cells of APHC patient samples (Fig. 2e; Supplementary Fig. S2b). Further data analysis revealed that tumor cells co-expressing HLA-DRA and CD74 might activate T cells and induce PD-(L)1 expression, compared to HLA-DRA-negative tumor cells (Supplementary Fig. S2c), indicating a more frequent interaction between T cells and malignant cells in APHC than in ANHC.

GSVA revealed that cell cycle-related pathways (e.g., MYC target and G2/M checkpoint), immune response pathways (e.g., IL-2 STAT5 signaling and TNF signaling via NF-κB), and aberrantly activated pathways (e.g., mTORC1 and PI3K/AKT/mTOR signaling) were dominant in tumor cells of APHC. In contrast, genes upregulated in ANHC were mapped to metabolic pathways (e.g., bile acid metabolism, fatty acid metabolism, and cholesterol homeostasis), which suggests that normal hepatic function might be partly preserved in ANHC (Supplementary Fig. S2d). Transcription factors (TFs) often work in combination to coordinate gene expression levels. Consistent with the results of GSVA, APHC contained regulators like the well-known proto-oncogene, MYC, and a vital interferon regulatory factor, IRF7. For ANHC, we found RXRA, retinoic acid X receptor α, FOXA2, and Forkhead Box A2, all of which played a key role in metabolism (Fig. 2f). According to previous studies^6^, AFP expression shared a high correlation with stemness feature in HCC. Therefore, the stemness of tumor cells was evaluated at a single-cell level, and the results were similar to the previous research (Supplementary Fig. S2e). Moreover, highly expressed genes from two groups were obtained and patients with high APHC signature scores had a poorer prognosis than patients with a low score in the Cancer Genome Atlas Liver Hepatocellular Carcinoma (TCGA-LIHC) cohort (Supplementary Fig. S2f). Histological grading (total *n* = 334; ANHC, *n* = 94; APHC, *n* = 240) also confirmed that APHC consisted of more moderate and poor differentiation samples (Supplementary Fig. S2g). Overall, our findings indicate that a hierarchical regulatory network works together to shape a more immunogenic and malignant feature in APHC tumor cells.

### Impaired T cell function in APHC

**Fig. 3:**
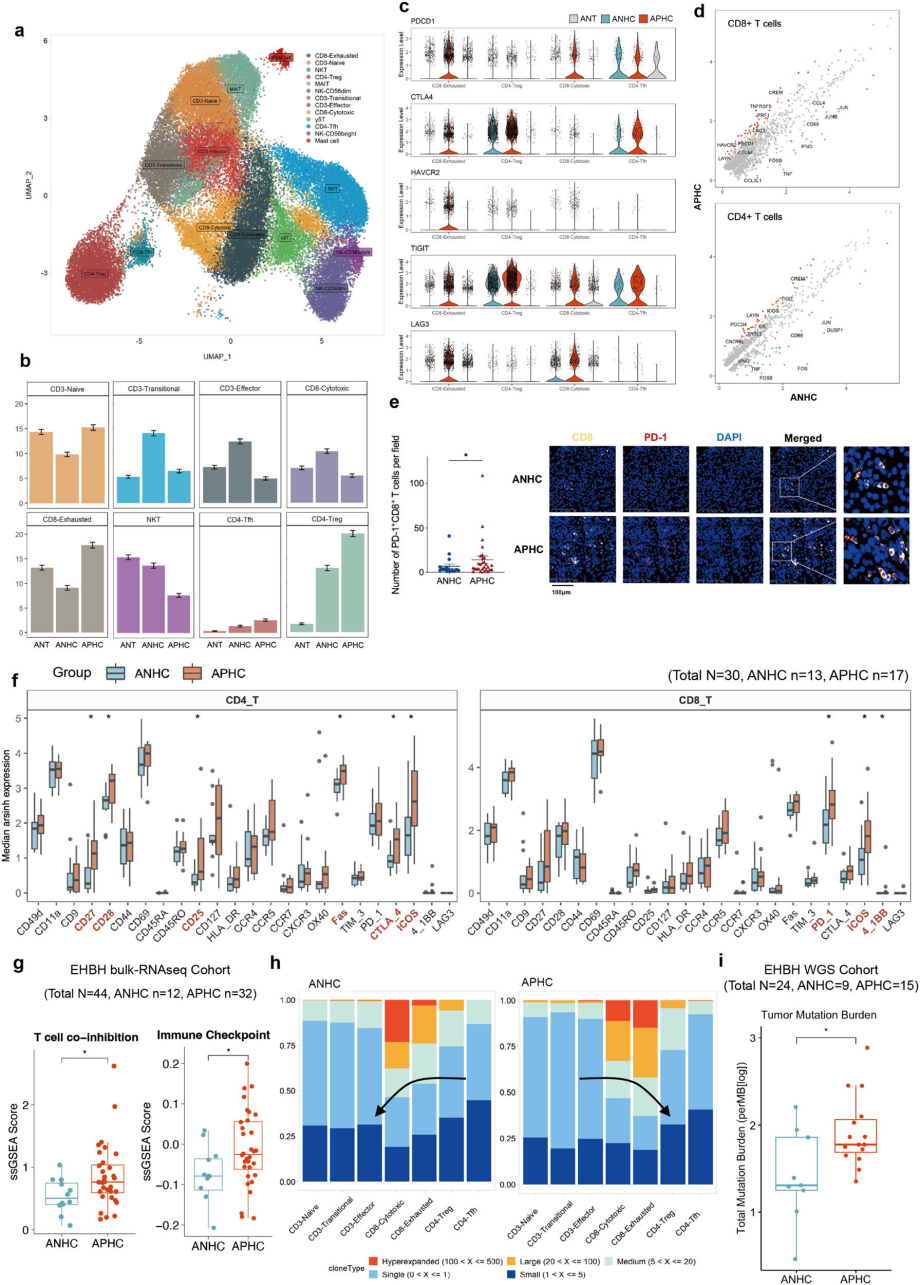
Landscape of tumor-infiltrating T cells in ANHC and APHC. **a** UMAP plot showing 13 cell subsets of T/NK cells. **b** Fractions of each T/NK subset across the groups of ANT, ANHC, and APHC. **c** Violin plots showing the expression level distribution of ICI-related genes. **d** Scatter plots showing the significantly differentially expressed genes of CD8^+^ and CD4^+^ T cells from APHC and ANHC. **e** The proportions of CD8^+^PD-1^+^ T cells between each group and the corresponding representative images. **f** Relative expression levels of each functional marker of CD4^+^ T cells and CD8^+^ T cells across recruited CyTOF cohort. *P < 0.05 using a Wilcoxon test. **g** Boxplots showing the expression levels of T cell immunity-related signatures scored by ssGSEA within the EHBH bulk RNA-seq cohort. **h** Clonal expansion status of T cell subsets across groups and decrease of TCR richness as the arrow indicated. **i** Boxplots showing TMB levels within the EHBH WGS cohort.

Graph-based clustering was used to analyze 52,723 T/NK cells, resulting in the identification of 9 broad categories of conventional (CD3^+^, CD4^+^, and CD8^+^) and 3 unconventional T cells (γδT, MAIT, and NKT cells), as well as two populations of innate lymphocytes comprising NK cells and mast cells that closely clustered with T cells (Fig. 3a; Supplementary Fig. S3a).

A higher diversity of T cells was observed in tumors than in adjacent nontumor tissues and functional heterogeneity of T cell populations was pronounced in both tumor groups. While the proportion of antitumor cells like CD3-Effector and CD8-Cytotoxic increased in adjacent non-tumor tissues, Tregs and CD8-Exhausted were enriched in tumor tissues (Fig. 3b). Over the past decade, several promising immune checkpoint inhibitors (ICIs) have demonstrated certain activity in clinical trials. Overall survival was slightly better among Asian patients with AFP levels of > 200 ng/mL in the KEYNOTE-240 trial (pembrolizumab vs placebo)^21^ and CheckMate 459 trial (nivolumab vs sorafenib)^22^. Remarkably, a continuum of ICI-related gene (*PDCD1*, *CTLA4*, *HAVCR2*, *TIGIT*, and *LAG3*) expression levels were observed, with the low expression on average in ANT, intermediate expression in ANHC, and high expression in APHC among four subsets of CD4^+^ and CD8^+^ T cells (Fig. 3c). Further analysis illustrated that exhaustion markers such as LYAN and TNFRSF9 were expressed in CD8^+^ T cells and TIGIT in CD4^+^ T cells of APHC, while effector genes like *IFNG* and *TNF* were relatively dominant in T cells of ANHC (Fig. 3d). These different gene expression patterns suggested that APHC was more likely to display an exhausted anti-tumor immune environment.

Next, we explored dynamic immune states and cell transcriptions in HCC-infiltrating CD8^+^ T cells. This analysis showed that T cells shared similar transition trajectories but resided in different states in ANHC and APHC. This transition was determined to initiate from CD3-Naive and CD3-Effector cells, through an intermediate transitional state and a cytotoxic state characterized by CD3-Transitional and CD8-Cytotoxic cells, to an exhausted state characterized by CD8-Exhausted cells (Supplementary Fig. S3b). Surprisingly, early-stage CD8^+^ T cells and terminally exhausted CD8^+^ T cells were predominantly distributed in APHC, whereas T cells at the transitional and the effector states were mainly observed in ANHC (Supplementary Fig. S3b, c). Moreover, marker gene trajectory analysis confirmed a more severe terminal differentiation state of T cells in APHC (Supplementary Fig. S3d).

To corroborate these findings from scRNA-seq at the protein level, we performed multiplex IHCs on a tissue microarray consisting of 45 HCCs (ANHC, *n* = 17; APHC, *n* = 28). Our data revealed a higher distribution of CD8^+^PD-1^+^ T cells, Tregs, and CD4^+^CTLA4^+^ T cells in the APHC group as compared to the ANHC group (Fig. 3e; Supplementary Fig. S3e, f). Furthermore, we developed a mass CyTOF panel incorporating 34 markers covering the phenotype of T cells (Supplementary Table S5). Tumor-infiltrating lymphocytes from additional ANHC (*n* = 13) and APHC (*n* = 17) samples were analyzed via CyTOF. Four major clusters of T cells based on protein expression patterns were identified (Supplementary Fig. S3g). Both CD4^+^ T cells and CD8^+^ T cells exhibited T cell dysfunction with the increased expression of various immune checkpoint molecules (i.e., CTLA-4 and PD-1) in APHC (Fig. 3f), while the distribution of double-positive T cells (DPT, CD3^+^CD4^+^CD8^+^) and double-negative T cells (DNT, CD3^+^CD4^–^CD8^–^) showed no difference between ANHC and APHC (Supplementary Fig. S3h). Next, single-sample Gene Set Enrichment Analysis (ssGSEA) on bulk RNA-seq data (total *n* = 44; ANHC, *n* = 12; APHC, *n* = 32) was conducted, and the data showed that APHC displayed a higher enrichment score in T cell exhaustion (Fig. 3g) and trafficking signature genes (Supplementary Fig. S3j). Collectively, our results indicated that T cells in APHC might have limited anti-tumor function.

We further assessed the T cell clonal expansion of 7 conventional T cell clusters from tumors which showed an aggregative distribution, indicating transcriptional heterogeneity in APHC and ANHC (Supplementary Fig. S3k). Moreover, > 50% of CD8-Exhausted T cells from APHC belonged to expanded clones, showing a higher amplification (> 5 clonotypes), whereas the expanded T cells from ANHC were mainly composed of CD8-Cytotoxic and CD8-Effector cells (Fig. 3h). Given that tumor mutation burden (TMB), neoantigen production, and T cell expansion were positively correlated^23^, we performed WGS analysis on 24 HCCs. From our data, APHC displayed a higher level of TMB, which might contribute to T cell activation and expansion, particularly in exhausted T cells (Fig. 3i). In summary, T cells in APHC inclined to immune dysfunction, which might be associated with more vigorous tumor immune evasion.

### AFP is involved in the immunomodulation of T cells in HCC

Based on previous findings, we wonder whether AFP is involved in regulating tumor immune evasion. Afp was knocked down in wild-type (WT) murine hepatocellular carcinoma cells (Hepa1-6) by short hairpin RNA (shRNA) interference (Hepa1-6 shAfp), with nontargeting construct as control (Hepa1-6 Control). Afp mRNA and proteins levels were notably downregulated in Hepa1-6 shAfp cells (Supplementary Fig. S4a, b). In vitro, there were no significant differences in cell proliferation between Hepa1-6 Control and Hepa1-6 shAfp (Supplementary Fig. S4c), ruling out the direct effect of Afp on cell proliferation.

**Fig. 4:**
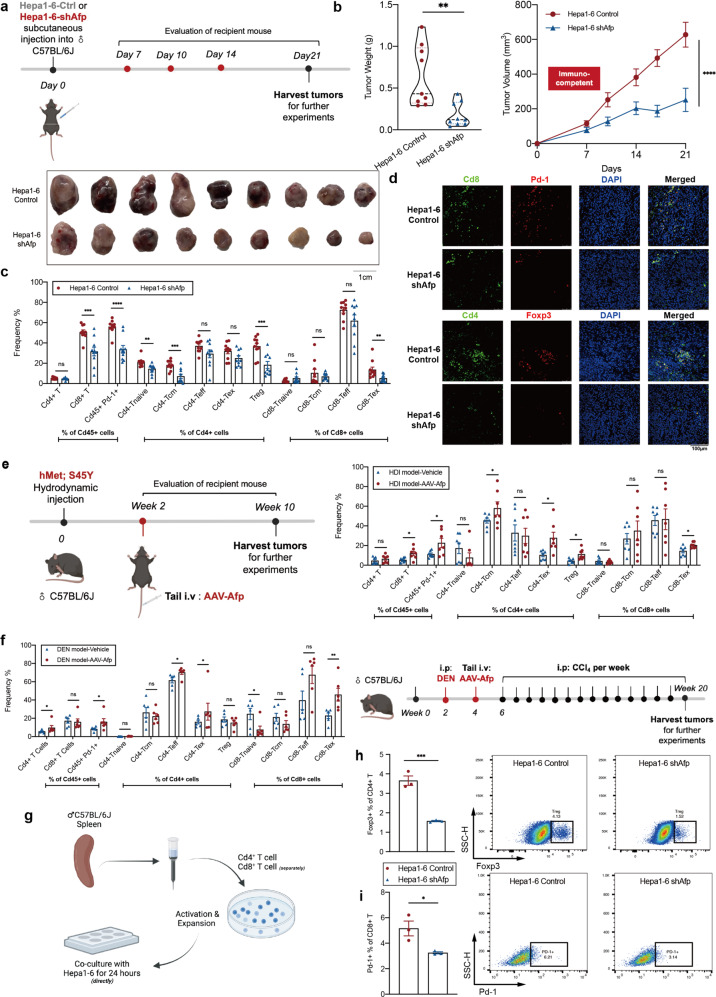
Afp was possibly involved in modulating the immune microenvironment of HCC. **a** Scheme showing the experimental procedure of the subcutaneous transplantation model. **b** Tumor weight and tumor growth curves in immuno-competent C57BL/6 mice injected subcutaneously with Hepa1-6-Ctrl or Hepa1-6-shAfp cells for 3 weeks. **c** Frequencies of corresponding T cell subsets analyzed by FACS in the subcutaneous transplantation model. **d** Representative multiplex IHC staining of Cd8^+^Pd-1^+^ T cells and Tregs (CD4^+^Foxp3^+^) in the subcutaneous transplantation model. **e** Scheme depicting the experimental procedure (left) and frequencies of corresponding T cell subsets analyzed by FACS (right) in the driver gene-induced HCC model by hydrodynamic tail vein injection. **f** Scheme presenting the experimental procedure (right) and frequencies of corresponding T cell subsets analyzed by FACS (left) in the DEN plus CCl_4_-induced HCC model. **g** Scheme presenting the experimental procedure of in vitro experiments. **h** Representative data of the frequency of Treg cells in the indicated groups by FACS. **i** Representative data of the frequency of Cd8^+^Pd-1^+^ cells in the indicated groups by FACS.

To assess liver cancer growth in vivo, Hepa1-6 Control or Hepa1-6 shAfp cells were inoculated subcutaneously into the flanks of immunodeficient mice (Nude mice). All mice developed tumors of comparable size on day 14 (Supplementary Fig. S4d, e). However, when the same experiment was conducted in immunocompetent C57BL/6 mice, mice injected with Hepa1-6 shAfp cells developed significantly smaller tumors than mice injected with control cells (Fig. 4a, b). Serum Afp level in Hepa1-6 shAfp group was notably decreased compared to that in the control group, but higher than that in mice without HCC cell inoculation (Supplementary Fig. S4h). The different outcomes of the two models indicated that Hepa1-6 shAfp and the control cells responded disparately to the anti-tumor immune environment. Subsequent immune cell composition analysis of xenografts formed in C57BL/6 mice showed a significant Cd8^+^ T cell infiltration in tumors of control groups, whereas the percentages of Cd4^+^ T cells were almost equivalent between the two groups. Nevertheless, the exhausted immune cells (Cd45^+^Pd-1^+^), Cd8-Tex, and Treg cells were more abundant in tumors formed with Hepa1-6 control. In addition, more infiltration of naïve Cd4^+^ T cells (Cd44^–^Cd62l^+^) and central memory Cd4^+^ T cells (Cd44^+^Cd62l^–^) were detected in the Hepa1-6 control group (Fig. 4c). Immunofluorescent labeling further demonstrated a higher proportion of Cd8-Tex and Treg cells within the tumor microenvironment with higher Afp level (Fig. 4d).

To characterize the immune response in terms of different Afp levels with the orthotopic model, concomitantly expressed hMet and an S45 to tyrosine mutant form of β-catenin (S45Y-βcatenin) were introduced via hydrodynamic tail vein injection^24^. Afp was overexpressed with i.v. adeno-associated virus (AAV) injection two weeks later (Fig. 4e). At the time of sacrifice, macroscopic white, cyst-like lesions were observed in the control group, while more liver tumors were formed in AAV-Afp treated group (Supplementary Fig. S4f). Flow cytometry analysis revealed that Cd8^+^ T cells, Cd4-Tcm, Cd4-Tex, Cd8-Tex, and Treg cells were accumulated in tumors with Afp overexpression (Fig. 4e). We further explored the role of Afp in the DEN plus carbon tetrachloride (CCl_4_)-induced HCC model, which mimicked the progression from chronic liver disease to HCC according to the previous report^25^. As expected, more and larger tumors were observed in the AAV-Afp group (Supplementary Fig. S4g). In this model, we also found that exhausted T cells and Treg cells were more abundant in the livers of the AAV-Afp group (Fig. 4f). In both models, serum AFP levels were also elevated after the injection of AAV-Afp (Supplementary Fig. S4i). Taken together, the data from the above animal models suggested that T cell function was more suppressive in Afp-overexpressing tumors.

Next, we performed in vitro functional assays to validate the role of Afp in modeling T cell function. Cd8^+^ T and Cd4^+^ T Cells were obtained from the spleen of C57BL/6J mice and then co-cultured with Hepa1-6 control or Hepa1-6 shAfp cells for 24 h after activation and expansion (Fig. 4g). Strikingly, we found that the proportion of Treg (Fig. 4h) and Pd-1^+^Cd8^+^ T cells (Fig. 4i) declined in Hepa1-6 shAfp co-cultured Cd4^+^ T cells and Cd8^+^ T cells, respectively.

These data provided evidence for the role of AFP in modulating T cell function, including but not limited to the recruitment of Treg and exhausted Cd8^+^ T cells, facilitating tumor cell survival.

### TAM-SPP1^+^ is elevated in TIME of APHC

**Fig. 5:**
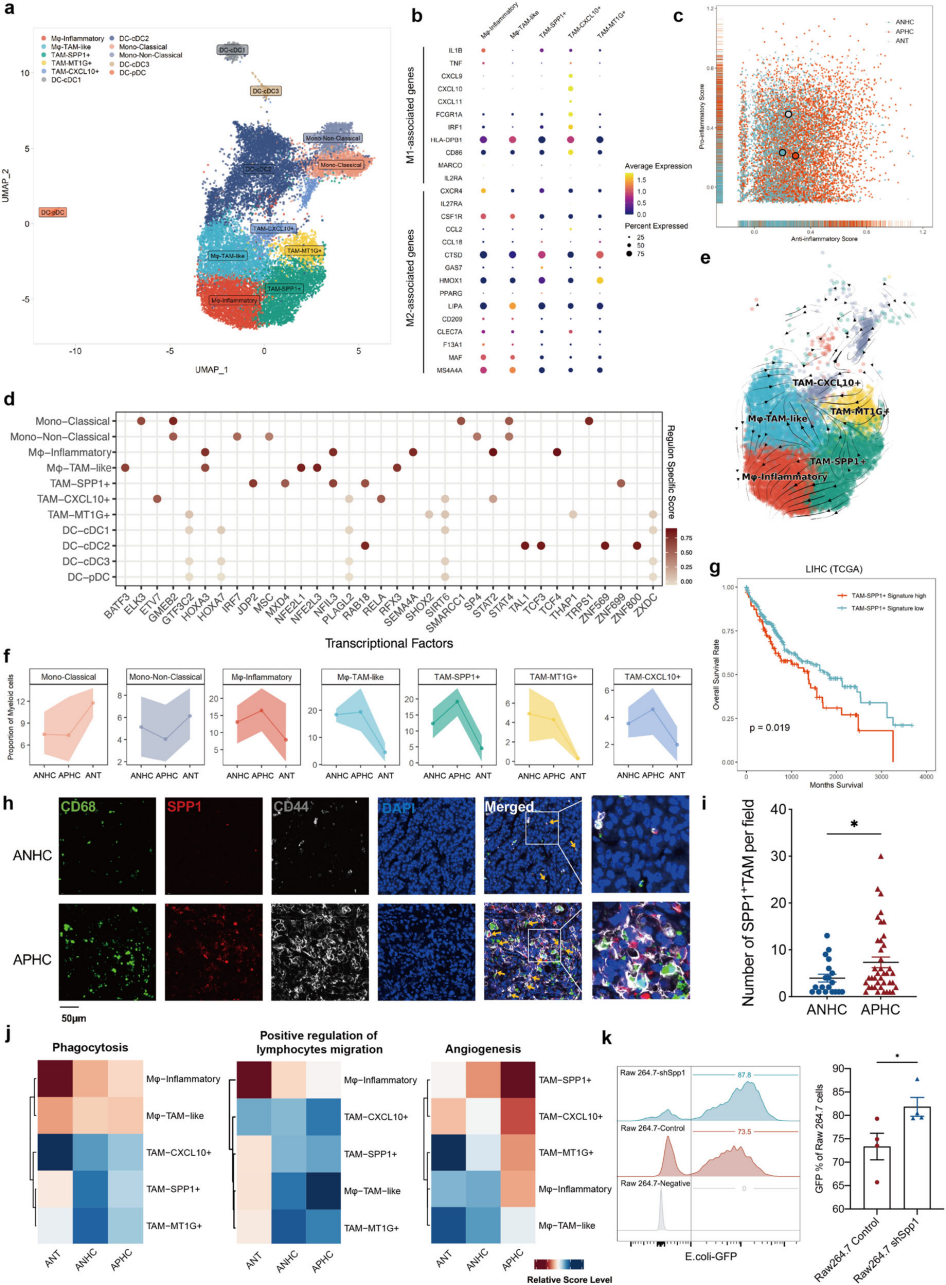
TAM-SPP1^+^ was enriched in APHC. **a** UMAP plot showing sub-clustering of myeloid cells identified monocyte, TAM, and DC. **b** Average expression of M1- and M2-like macrophage-related genes in 5 subsets of macrophages. **c** Pro- and anti-inflammatory scores for macrophages in ANT, ANHC, and APHC. **d** Predicted transcriptional factors of each myeloid subpopulation. **e** RNA velocity of 5 subsets of macrophages. **f** Fractional changes for each monocyte and macrophage cell type in ANT, ANHC, and APHC. **g** Kaplan-Meier plots of overall survival in patients from TCGA dataset defined by TAM-SPP1^+^ signature. **h** The multiplex IHC staining of TAM-SPP1^+^ (CD68^+^SPP1^+^) and CD44 in ANHC and APHC (total *n* = 59; ANHC, *n* = 19; APHC, *n* = 40). **i** The average number of TAM-SPP1^+^ cells per field in ANHC and APHC. **j** Heatmaps showing different expression patterns of function-associated signature genes among 5 macrophage subsets in ANT, ANHC, and APHC. **k** Scavenger function of Raw 264.7 control compared with Raw 264.7 shSpp1 analyzed by FACS.

Next, a total of 11 clusters emerged within the myeloid lineage, comprising 2 clusters for monocytes, 5 for macrophages, and 4 for DCs (Fig. 5a). The distinct clusters were determined by their specific gene expressions and known lineage markers (Supplementary Fig. S5a). Notably, a simple M1/M2 dichotomization of macrophages was undesirable for capturing the diversity of TAM phenotypes (Fig. 5b). Instead, by utilizing signature genes of M1/M2 in macrophage subsets, we observed that only Mφ-Inflammatory displayed the highest pro-inflammatory score, and three TAMs exhibited lower M1 signatures (Supplementary Fig. S5c). Furthermore, the overall functional phenotypes of macrophages were analyzed and increased anti-inflammatory features in APHC were revealed (Fig. 5c). To confirm the extensive plasticity and potential cell fate conversion between the subgroups of myeloid cells, the single-cell regulatory network inference and clustering (SCENIC) and RNA velocity method were employed, which identified the TFs involved (Fig. 5d, e).

According to the results, macrophage clusters were mostly exhibited in tumors, while DCs (Supplementary Fig. S5b) and monocytes were enriched in normal tissue (Fig. 5f). Notably, among the TAM subpopulations, TAM-CXCL10^+^ and TAM-SPP1^+^ were highly enriched in APHC compared to ANHC, and the latter TAM subset has been frequently observed in various cancer research^26,27^. Therefore, we further analyzed TAM-SPP1^+^, a unique subpopulation recently reported in colorectal cancer (CRC) and metastatic tissue^26,28^. We first integrated the dataset of CRC from Qi et al.^27^ with our data. The functions of TAM-SPP1^+^ in HCC and CRC were potentially similar; however, TAM-SPP1^+^ in HCC appeared to exhibit an anti-inflammatory phenotype (Supplementary Fig. S5d, e). Importantly, we found that TAM-SPP1^+^, characterized by the highest M2 signature, was associated with poor patient prognosis in the TCGA RNA-seq cohort of HCC (Fig. 5g). The distribution of TAM-SPP1^+^ in APHC and ANHC (Total *n* = 59; ANHC, *n* = 19; APHC, *n* = 40) was further confirmed by multicolor IHC (Fig. 5h, i).

SPP1 has been previously implicated in hypoxia conditions and promotes fibrosis in various diseases^29,30^. We next used knnDREMI to classify two gene modules that corresponded to SPP1 within TAM, marking high corresponding genes in the red box and low corresponding genes in the black box (Supplementary Fig. S5f). Candidate genes in the high module included GPI (Glucose-6-phosphate isomerase), ALDOC (Aldolase C, fructose-bisphosphate), and GAPDH (Glyceraldehyde 3-phosphate dehydrogenase), which were among the enriched pathways related to glycolysis (Supplementary Fig. S5g). We also identified vascular endothelial growth factor A (VEGFA), a cytokine induced in low-oxygen conditions, positively correlated with SPP1 and the increase of GPI level (Fig. 5h).

To explore the biological function of the TAM-SPP1^+^ subset, we assessed phagocytosis, capacity to regulate lymphocyte migration, and angiogenesis properties. Our findings indicated that TAM-SPP1^+^ exhibited a low phagocytosis score, reduced ability to regulate lymphocyte migration, and the highest angiogenesis score (Fig. 5j), consistent with previous observations^31^.

We further investigated the function of TAM-SPP1^+^ by knocking down SPP1/Spp1 in THP-1 and Raw 264.7 cell lines, respectively (Supplementary Fig. S5i, j). After the knockdown of SPP1/Spp1, Raw 264.7 cells were more likely to polarize into the M1-state and showed an enhanced ability to engulf *Escherichia coli* (Fig. 5k; Supplementary Fig. S5k). We also examined the role of TAM-SPP1^+^ in HUVEC tube formation and recruitment. The angiogenesis of HUVEC was weaker when exposed to the conditional medium (CM) from THP-1 shSPP1 compared to the CM from THP-1 CTRL (Supplementary Fig. S5l, m). Taken together, a specific subset of TAM, TAM-SPP1^+^ was enriched in APHC, showing weakened phagocytosis but enhanced angiogenesis capacity and lymphocyte migration.

### TAM-SPP1^+^ plays a role in T cell exhaustion in APHC

**Fig. 6:**
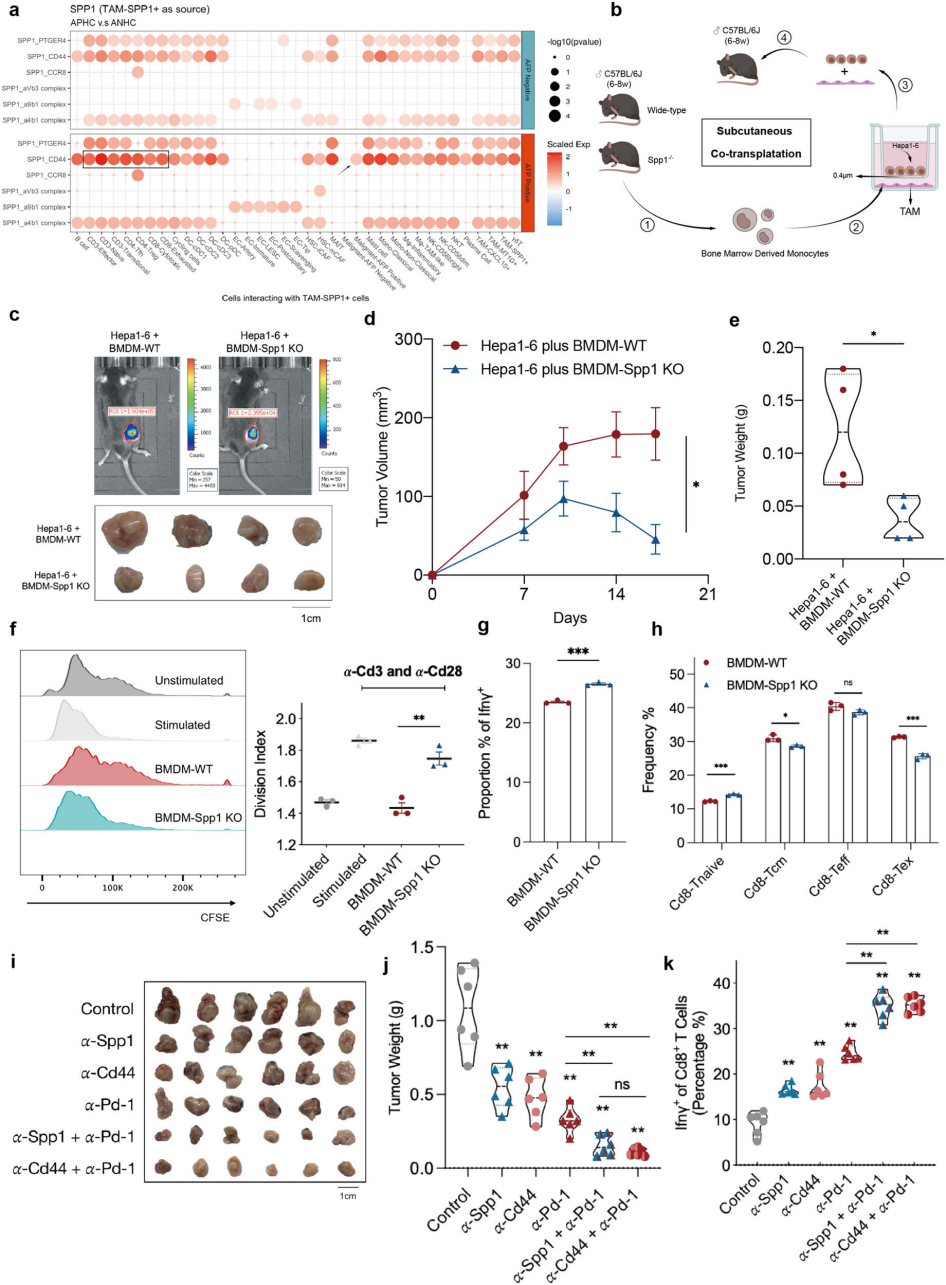
SPP1-CD44 signaling axis promoted tumor progression and impaired T cell immunity. **a** Dot plot showing receptor–ligand pair analysis of the interactions between TAM-SPP1^+^ and distinct cell types. **b** Scheme presenting the experimental procedure of the subcutaneous co-transplantation model. **c** Luminescence imaging of mice and gross morphology of tumors in the subcutaneous BMDM and Hepa1-6 co-transplantation model. BMDMs were derived from WT C57BL/6J and Spp1^–/–^ C57BL/6J mice, respectively. **d** Tumor growth curves of the subcutaneous co-transplantation model. **e** Tumor weight in the subcutaneous co-transplantation model. **f** CFSE intensity of T cell after co-culturing with BMDM-WT or BMDM-Spp1 KO was quantified as a division index (BMDMs were introduced to exhibit an M2 phenotype before co-culture). **g** The proportion of Ifn-γ of Cd8^+^ T cells co-cultured with the indicated BMDM derived from WT C57BL/6J and Spp1^–/–^ C57BL/6J mice, respectively. **h** The frequency of different subsets of T cells from the in vitro model was quantified by FACS. **i** Gross morphology of tumors in the C57BL/6 mice administered with in vivo therapies. **j**, **k** Tumor weight (**j**) and frequencies of Ifn-γ^+^Cd8^+^ T cell (**k**) of C57BL/6 mice implanted with Hepa1-6 and then administered with different therapies (α-Spp1, anti-Spp1 antibody; α-Pd-1, anti-Pd-1 antibody; α-Cd44, anti-Cd44 antibody).

To decipher the interactions between TAM-SPP1^+^ and other subpopulations, we conducted the analysis using CellPhoneDB to explore possible interplaying ligands and receptors. Specifically, SPP1 was still the top-ranked ligand, which could bind to integrins, Prostaglandin E2 receptor 4 (EP4), Chemokine (C-C motif) receptor 8 (CCR8), and CD44 (Fig. 6a). CD44 was the most widely known receptor of SPP1, mainly found on T cells (black box) and tumor cells (black arrow). Notably, CD44, a stem-like marker, was expressed only in malignant cells of APHC other than ANHC, which also validated a more stem-like feature of APHC. Further, in the aforementioned multiplex IHC staining of TAM-SPP1^+^, CD44 appears to be positively correlated with the abundance of TAM-SPP1^+^ (Fig. 5h). As expected, due to the ligand–receptor feedback mechanism, when HCC cell lines, Huh7, PLC, and MHCC-LM3 were co-cultured with THP-1 CTRL or THP-1 shSPP1 in vitro, respectively, CD44 levels declined in THP-1 shSPP1 co-cultured cells (Supplementary Fig. S6a, b).

CD44 is also a prominent activation marker that distinguishes memory and effector T cells from their naïve counterparts; thus, we hypothesized that TAM-SPP1^+^ could also facilitate tumor cell evasion by suppressing CD8^+^ T cell activation. To investigate whether TAM-SPP1^+^ could promote cancer cell survival via mediating TIME, Hepa1-6 and Spp1-KO bone marrow-derived macrophage (BMDM) from Spp1^–/–^ mice^32,33^ or control BMDM from C57BL/6J mice were mixed and implanted subcutaneously to establish a tumor-bearing model (Fig. 6b, details in Materials and Methods). Tumors grew slower and smaller in the Hepa1-6 plus Spp1^–/–^ mouse-derived BMDM implanted group than in the control group (Fig. 6b–e). As expected, a relatively higher proportion of suppressive T cells and TAMs was observed in Hepa1-6 formed tumors in BMDM-Spp1 KO group as indicated by multiplex IHC, suggesting the role of Spp1 in modulating TIME (Supplementary Fig. S6c, d).

Furthermore, we cultured pre-activated Cd8^+^ T cells harvested from the spleen of WT mice with BMDM-WT or BMDM-Spp1 KO in vitro (Supplementary Fig. S6a). The immunological assessment revealed that BMDM-WT exerted a suppressive effect on the proliferation capacity of Cd8^+^ T cells (Fig. 6f) as well as Ifn-γ production compared to BMDM-Spp1 KO (Fig. 6g; Supplementary Fig. S6e). Moreover, Cd8^+^ T cells cultured with BMDM-WT tended to exhibit a memory/exhausted phenotype (Fig. 6h), suggesting the altered T cell conversion and function of BMDM with different Spp1 levels.

The above findings led us to investigate whether impairing the Spp1-Cd44 axis via mono-antibody blockade could enhance the efficacy of anti-Pd-1 therapy. In Hepa1-6 implanted tumor-bearing model, anti-Spp1 monoclonal antibody (mAb), anti-Cd44 mAb, or anti-Pd-1 mAb alone significantly inhibited the growth of tumors. Nevertheless, combination therapy exhibited a synergistic effect on the inhibition of tumor growth (Fig. 6i, j; Supplementary Fig. S6f). Further analysis revealed that blockage of the Spp1-Cd44 axis combined with anti-Pd-1 led to higher Ifn-γ^+^Cd8^+^ T cell production than treatment with a single mAb (Fig. 6k). Thus, our results demonstrated the role of TAM-SPP1^+^ in facilitating an evasive TIME, which could be hindered by targeting Spp1-Cd44 axis.

### Upregulation of TAM-SPP1^+^ via TGF-β1 and IL6 signaling

To further clarify the origin of TAM-SPP1^+^, we focused on stromal cells (i.e., endothelial cells (ECs), fibroblasts) since previous studies indicated that SPP1 was involved in multiple fibrotic diseases^34,35^. The subpopulations of stromal cells were clustered based on the expression of canonical gene signatures (Supplementary Fig. S6g, h). Especially, two tumor-specific cells, including EC-immature and EC-Tip cells were identified; both highly expressed plasmalemma vesicle-associated proteins (PLVAP) indicative of the presence of endothelial fenestrations and were enriched in similar biological pathways (Supplementary Figs. S6i, j, S7a–d). Tip cells, expressing gene signatures associated with matrix remodeling like lysyl oxidase (LOX) under control of VEGF signaling, were abundant in tumors, especially in APHC, which was confirmed by immunofluorescence (Supplementary Figs. S6i, S7b).

**Fig. 7:**
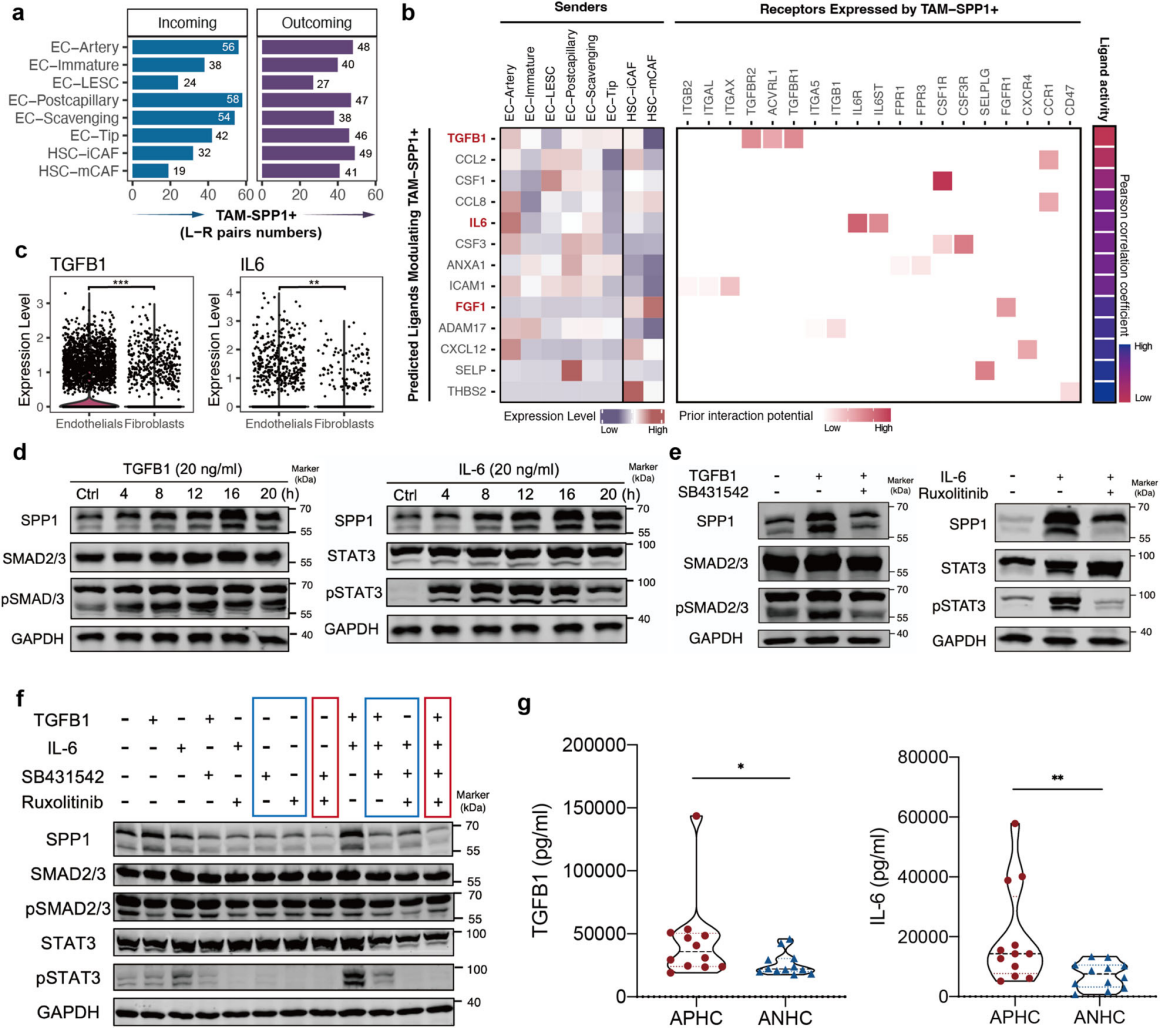
Excessive TGF-β1 and IL6 signaling activation promoted the enrichment of TAM-SPP1^+^ in APHC. **a** Bar plot presenting the numbers of putative ligand–receptor pairs between stromal cells and TAM-SPP1^+^ using scRNA-seq data. **b** Left, heatmap of relative expression across subpopulations of the top predicted ligands expressed by stromal cells using scRNA-seq. Middle, heatmap of significant ligand–receptor pairs between ≥ 1 stromal cell subsets and TAM-SPP1^+^ in scRNA-seq. Right, top predicted ligand colored by activity. **c** Violin plots showing the expression of TGF-β1 and IL6 in stromal cells. **d** Western blot analysis of protein expression induced by TGF-β1 (left) or IL6 (right) in THP-1 under the stimulation of PMA at the indicated time points. **e** Western blot analysis of protein level in PMA-stimulated THP-1 treated with SB431542 (10 μM, left) or Ruxolitinib (10 μM, right) with or without TGF-β1 (20 ng/mL, left) or IL6 (20 ng/mL, right) for 16 h, respectively. **f** Western blot analysis of protein level with mono treatment or combined treatment of TGF-β1, IL6, and their corresponding inhibitors (TGF-β1, 20 ng/mL; IL6, 20 ng/mL; SB431542, 10 μM; Ruxolitinib, 10 μM). **g** The concentrations of TGF-β1 and IL6 from homogenates of additional APHC (*n* = 12) and ANHC (*n* = 12) patient tissues measured by ELISA.

Next, the cell–cell interaction inferred by ligand–receptor analysis indicated abundant intercellular communication between stromal cells and TAM-SPP1^+^. Specifically, prominent stromal cell signaling to TAM-SPP1^+^, which could induce SPP1 expression, was mediated by TGF-β1, IL6, and FGF1 (Fig. 7a, b; Supplementary Fig. S7e). By integrating the result of CellPhoneDB (Supplementary Fig. S7f), we finally identified two candidate molecules, TGF-β1 and IL6, that could induce SPP1 expression in macrophages (Fig. 7c).

Herein, in vitro study showed that IL6 and TGF-β1 can both upregulate TAM-SPP1^+^ in a time-dependent manner in differentiated THP-1 (Fig. 7d). However, the two molecules did not elevate SPP1 in a dose-dependent manner (Supplementary Fig. S7g). Consistently, the induction of SPP1 was abrogated by TGF-β1/SMAD inhibitor, SB431542, and IL6/STAT3 inhibitor, Ruxolitinib (Fig. 7e). Notably, combined treatment of both SB431542 and Ruxolitinib (marked in red) exerted the most intensive inhibition of SPP1 expression in THP-1 than SB431542 or Ruxolitinib alone (marked in blue), which indicated that THP-1 maintained a certain level of TGF-β1 and IL6 secretion, and activation of either pathway could upregulate the proportion of TAM-SPP1^+^ (Fig. 7f).

To validate whether TGF-β1 and IL6 contribute to the emergence of TAM-SPP1^+^ in HCC, we further measured concentrations of TGF-β1 and IL6 in HCC homogenates of APHC (*n* = 12) and ANHC (*n* = 12). The level of TGF-β1 and IL6 positively correlated with each other (Supplementary Fig. S7h). Importantly, APHC showed a significantly higher level of TGF-β1 and IL6 than ANHC (Fig. 7g), which probably contributed to enhanced accumulation of TAM-SPP1^+^ and the subsequent exhausted anti-tumor immune state.

## Discussion

**Fig. 8:**
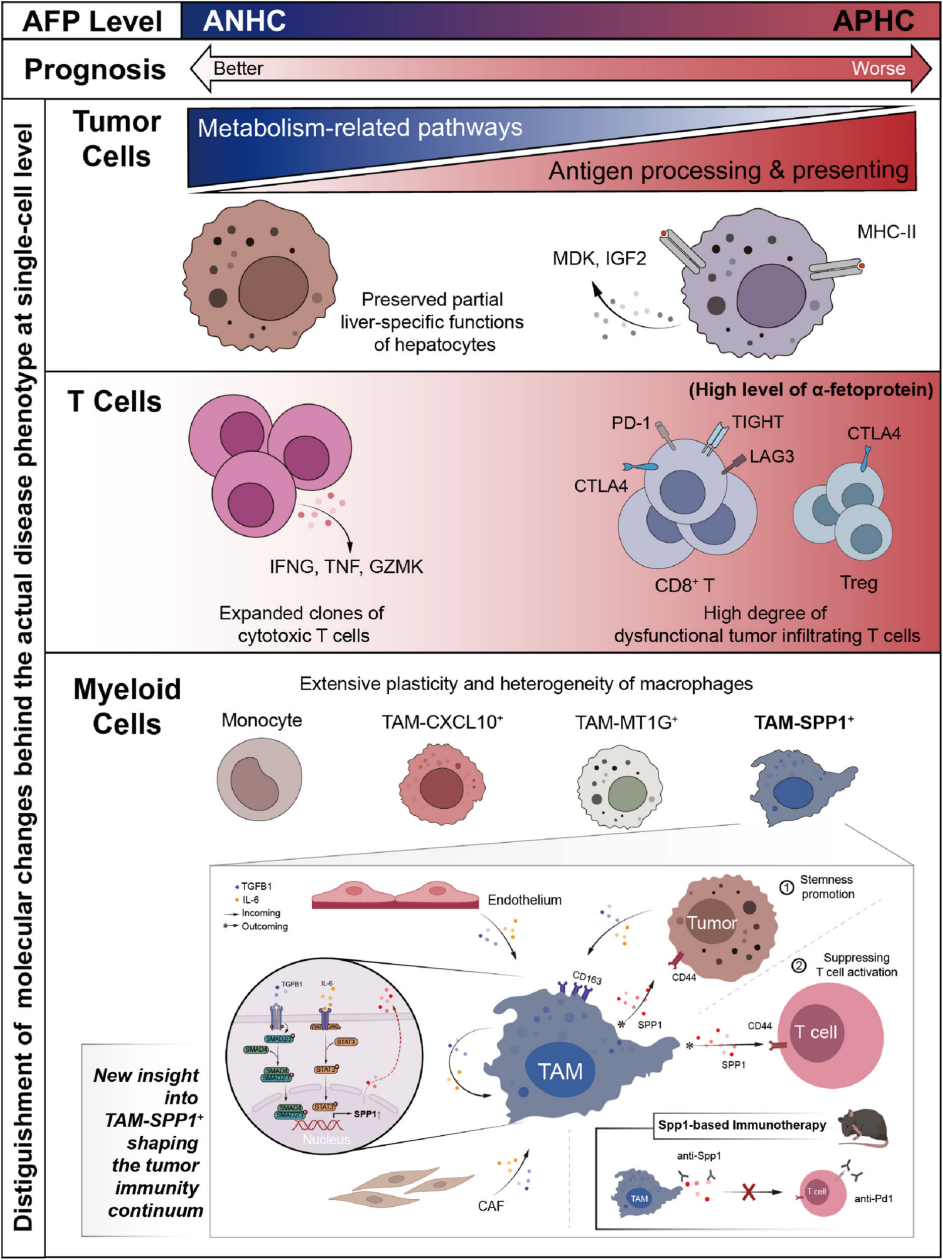
Summary of immune features and dynamics in ANHC and APHC. Graphical summary of immune microenvironment dynamics among ANHC and APHC.

Since the encouraging results of the IMbrave 150 trial^36^, immunotherapy has had unprecedented success in advanced HCC but still only substantively benefits a small proportion of patients. How to individualize immunotherapy in HCC patients to harness the host immune system against cancer cells has become a key question. Briefly, overall survival was slightly better among Asian patients with AFP levels of > 200 ng/mL in the CheckMate 459 trial^37^ and KEYNOTE-240 trial^21^. Ramucirumab, an inhibitor of the VEGF receptor 2, also showed efficacy after sorafenib treatment among patients with AFP levels > 400 ng/mL^38^. However, rare studies investigated the underlying mechanism related to the immune response of AFP-relevant HCC. In this study, we integrated multiple omics to decipher the pre-immunotherapy states of ANHC and APHC, which are required to identify new therapeutic strategies (Fig. 8).

First, we found that APHC had a worse prognosis and a spectrum of differences between cell lineages in ANHC and APHC as revealed by scRNA-seq. Intensive upregulation of antigen processing and presentation on tumor cells from APHC was confirmed, which is in line with previous findings showing that the AFP^high^ HCC samples upregulate immunity-related pathways^10^. Although HLA-DR^+^ tumor cells were not separated in vitro, we could not rule out the possibility that MHC-II molecules on tumor cells might react with T cells^19,39^. Similarly, the metabolic feature within ANHC is consistent with Ling’s findings^40^ that ANHC featured metabolic pathways. We hypothesized that this might be due to the better differentiation of ANHC, which maintained most of the normal functions of hepatocytes.

Next, we revealed diverse phenotypic and functional T cell states in both APHC and ANHC. APHC had increased suppressive T cells but decreased cytotoxic T cells; in addition, more ICI-related genes were expressed in T cells within APHC. By contrast, ANHC had more cytotoxic T subsets, resulting in higher expression of functional molecules. We further profiled T lineages in bulk RNA-seq and CyTOF cohort, which strongly confirmed that APHC exhibits more suppressive TIME. Besides, APHC presented high clonal expansion in CD8-Exhausted, while the cell compartment with clonally expanded TCR in ANHC was CD8-Cytotoxic. In light of MHC-II on the surface of tumor cells, our data suggested that more neoantigens within APHC as indicated by heavier TMB might have elicited the expansion of T cells.

Previous research provided evidence that AFP arbitrated immune escape in HCC by enhancing the apoptotic markers in infiltrating immune cells^41^. Pardee et al.^11^ reported that tumor-derived AFP (tAFP) served as a key regulator of DC differentiation, of which tAFP-conditioned DCs retained a naïve phenotype and produced limited levels of inflammatory cytokines and chemokines. These studies were mainly based on in vitro studies and a few murine models. Significantly, our study revealed a pro-tumor microenvironment in APHC and further proved that Afp-positive HCC could suppress T cell function both in vivo and in vitro.

The discovery of the heterogeneity and plasticity of TAM, especially the identification of TAM-SPP1^+^, is in accord with recent research. Zhang et al.^31^ reported that TAM-SPP1^+^ was associated with angiogenesis in CRC. A pan-cancer study of myeloid cells in TIME annotated this TAM subset across 8 tumor types^42^, of which TAM-SPP1^+^ exhibited the highest M2 signatures. scRNA-seq analysis of 6 cancer types revealed that SPP1^+^ TAM expanded in hypoxia and promoted cancer malignancy^43^. Specifically, recent studies of CRC highlighted the infiltration of SPP1^+^ macrophage, which correlated with FAP^+^ fibroblasts and predicted the metastasis of CRC to the liver^26–28^. Although these studies have reported a possible role of TAM-SPP1^+^, they did not fully characterize the complexity of TAM-SPP1^+^ interacting with other cells. We identified that the SPP1-CD44 axis sufficiently participated in immune suppression and tumor promotion. Impairing this axis relieved the exhausting phenotype of T cells, which motivated us to investigate the combination of anti-Spp1 mAb or anti-Cd44 mAb to remodel the TIME with anti-Pd-1 mAb for enhanced treatment efficacy of HCC. This finding suggested that inhibiting upstream signaling of T cells could alter immune suppression to achieve better tumor inhibition. Furthermore, we found a potential mechanism that TGF-β1 and IL6 signaling activation in APHC, most abundantly in stromal cells, induced the SPP1 expression in macrophages.

Our study has several limitations worth mentioning despite the above findings. First, the limited sample size in the scRNA-seq resulted in no statistical significance among the groups. Although we have validated our findings in a larger cohort in multiple dimensions, a larger cohort might depict the features underlying the high heterogeneity of HCC more comprehensively. Meanwhile, the difference between ANHC and APHC, especially the indication of altered immune response needs to be confirmed in larger clinical cohorts. Additionally, whether the immune suppression was relieved in APHC patients after surgery when AFP level declined remained unknown, which is worth exploring for the selection of subsequent immune therapy.

To conclude, our study provides novel insight into individualized therapy in HCC with different levels of AFP by integrating multi-omic analysis, enabling an understanding of the immunity continuum of HCC. Our work also provides additional comprehension into tumor biology that may help shape the TIME and informs novel therapeutic strategies for overcoming immune suppression.

## Materials and methods

### Participants

The tissue microarray composed of HCC samples was obtained from Eastern Hepatobiliary Surgery Hospital (EHBH), Shanghai, China (*n* = 185). HCC (*n* = 12) and patient-matched normal adjacent tissues (*n* = 4) were collected for scRNA-seq under a protocol. Additional 30 HCC specimens were stained with metal-labeled antibodies for CyTOF analysis. Individuals donating fresh surgical tissues provided informed consent. All diagnoses were verified by histological review by a board-certified pathologist.

The study was conducted in accordance with the principles of the Declaration of Helsinki and approved by the ethics committee of EHBH (EHBHKY2023-K012-P001). Informed written consent was obtained from all participants before being enrolled in the study.

### Mice

Constitutive Spp1-deficient mice in a C57BL/6J background were a kind gift of Dr. Jinhong Chen from Fudan University, Shanghai, China. WT male C57BL/6J mice were brought from the Nanjing Biomedical Research Institute of Nanjing University.

All mice used for the experiment in this study were males between 6 and 8 weeks old. The ethics committee of EHBH approved housing and all experimental animal procedures (IACUC Issue# EWDLL-003).

### Murine BMDMs

Macrophages were derived from bone marrow precursors as described before^44^. Briefly, bone marrow cells (1 × 10^7^ cells) were cultured in a volume of 6 mL in a 10 cm Petri dish (non-tissue culture treated, bacterial grade) in Dulbecco’s modified Eagle’s medium (DMEM) supplemented with 20% fetal bovine serum (FBS) and 100 ng/mL M-CSF. After 3 days in culture, the medium was supplemented with an additional 3 mL of the differentiation medium. On day 7, BMDMs were harvested with ice-cold Ca^2+^- and Mg^2+^-free PBS (DPBS). The cells obtained were mature macrophages as assessed by flow cytometry and then were collected for further experiments.

### Mouse models

For the subcutaneous model, 2 × 10^6^ Hepa1-6 shCtrl and shAfp cells were injected into the right thighs of C57BL/6 or nude mice. The number of mice per group for each experiment is detailed in the figure legends. Tumor size (length × width^2^ × 0.5) was measured twice per week after injection. The mice were euthanized around 3 weeks and the tumors were harvested for weight measurement and further tissue analyses. For the hMetS45Y-β-catenin and hMet model, hydrodynamic tail vein injections were performed. Briefly, 20 μg pT3-EF5a-hMet-V5 or pT3-EF5a-S45Y-β-catenin-Myc or the combination of pT3-EF5a-hMet-V5 and pT3-EF5a-S45Y-β-catenin-Myc, together with the sleeping beauty (SB) transposase were diluted in 2 mL of normal saline (0.9% NaCl) in a ratio of 25:1 and injected into the tail vein of 6–8-week-old C57BL/6 mice for 5–7 s. For a long-term model of HCC, 6-week-old male mice were injected with DEN (25 mg/kg; Sigma-Aldrich, St. Louis, MO, USA) intraperitoneally (i.p.) at the age of 15 days and a single injection of DEN was followed by administration of 1 mL/kg CCl_4_ per week. AAV-Vehicle (3 × 10^11^ v.g./mouse) or AAV-Afp was administered at the time point shown in the diagram.

1 × 10^6^ Hepa1-6 cells with 1 × 10^6^ BMDM-WT or BMDM-Spp1-KO were subcutaneously injected into the right side of the mice. After 3 weeks, mice were sacrificed for analysis of tumor formation.

For in vivo treatments, 2 × 10^6^ Hepa1-6 cells were subcutaneously injected into the right side of mice. A total of 100 μL PBS containing 100 μg immunoglobulin (Ig) or anti-Spp1 or anti-Pd-1 or anti-Spp1 plus anti-Pd-1 were i.p. injected once every two days.

### Cell–cell communication analysis

We employed a method called CellPhoneDB tailored for single-cell transcriptome data to explore cell–cell communications based on the manually curated repository of interacting ligands and receptors^45^. Briefly, the method infers potential cell–cell interactions based on the expression of interacting ligand–receptor pairs between two clusters. The gene encoding receptor or ligand included in the downstream analysis should be expressed by > 30% of cells in a specific cluster. To identify the significance of a ligand–receptor pair between two clusters, the permutation test was performed by randomly assigning the cluster labels of each cell 1000 times. An empirical *P*-value was determined by the rank of actual average expression of a given ligand and receptor pair in two clusters among the 1000 permutated results.

For NicheNet analysis^46^, we derived cell type signatures by taking the top DEGs (avg_Log2FC > 1) in cells isolated from tumors, including endothelium, fibroblasts, and TAM-SPP1^+^. We input these signatures into NicheNet to derive a union set of predicted ligands modulating tumor-specific TIME cell type signatures. For ligands predicting TAM-SPP1^+^ modulation, we input the top TAM-SPP1^+^ DEGs. The top 20% of predicted ligands by the regulatory potential that also demonstrated significance in our scRNA-seq ligand–receptor interaction analysis (described above) in each case is shown in Supplementary Fig. S7e.

### Transwell co-culture system

For tumor sphere formation and immune cell interaction with the Transwell co-culture system, confluent TAM-control or TAM-shSPP1/shSpp1 were placed in the upper insert (6.5 mm diameter with Polyethylene terephthalate membrane filters containing 0.4 μm pores, Corning, NY, USA). After induction, the inserts were moved into wells attached to Huh7 or preactivated T cells.

### Cell lines

The murine HCC cell line Hepa1-6, murine macrophage cell line Raw 264.7, and human HCC cell line Huh7, PLC3, and LM3 were purchased from the Cell Bank of Type Culture Collection of the Chinese Academy of Sciences (CBTCCCAS). The human monocytic cell line THP-1 was purchased from Shanghai Zhong Qiao Xin Zhou Biotechnology Co., Ltd.

Cell lines were authenticated by STR profiling and verified to be mycoplasma negative. Hepa1-6, Huh7, PLC, LM3, and Raw 264.7 cells were grown in DMEM (Gibco, USA), and THP-1 cells were grown in Roswell Park Memorial Institute (RPMI) 1640 Medium. All cell lines were cultured in a medium supplemented with 10% FBS, 100 units/mL penicillin, and 100 mg/mL streptomycin at 37 °C and 5% CO_2_.

### Transfection for the stable cell line

The lentivirus-mediated shRNA expressing vector targeting mouse Afp was purchased from Obio Technology (Shanghai, China). Cells (60%–70% confluency) were incubated in a medium containing optimal dilutions of lentivirus mixed with polybrene. After 48 h of transfection, cells were subjected to puromycin selection (5 mg/mL) to obtain the stably transfected cells.

The lentivirus specific to THP-1 and RAW 264.7 with shRNA targeting SPP1/Spp1 was purchased from Genechem Co., Ltd (Shanghai, China). The transfection was performed following the manufacturer’s instructions.

### Real-time quantitative PCR

Total cellular RNA was extracted using Trizol reagent (Invitrogen) and reversely transcribed into cDNA using Superscript III reverse transcriptase (Invitrogen) and random primers according to the manufacturer’s guidelines. The resulting cDNA was subsequently used as a template for the amplification of target gene transcripts by real-time PCR, using SYBR Green PCR Master Mix (Applied Biosystems) on ABI PRISM 7300HT Sequence Detection System (Applied Biosystems). β-actin was used as a control for normalization. The primers are listed in Supplementary Table S4.

### Flow cytometry

For murine T cell phenotype analysis, cells were stained with anti-Cd45, -Cd4, -Cd8, -Cd44, -Cd62l, and -Pd-1 for 30 min at 4 °C. For intracellular staining, T cells were stimulated in the presence of PMA (50 ng/mL), Ionomycin (1 μg/mL), and Brefeldin A (5 mg/mL) for 4–6 h. For intranuclear staining, cells were fixed and permeabilized with Transcription Factor Fixation/Permeabilization set for 40 min at room temperature, followed by anti-Foxp3/anti-Ifn-γ staining. All data were acquired immediately by an LSRFortessa flow cytometer (BD Biosciences) and analyzed using FlowJo software (Tree Star, Inc., Ashland, OR, USA).

### Cell proliferation assay

For cell proliferation assay, relevant cells were seeded in 96-well plates at a density of 4 × 10^3^ cells per well. The cell viability was determined by CCK-8 assay kit following the manufacturer’s instructions at the indicated time points after seeding. The results were analyzed using GraphPad Prism 8.0.

### Western blot

Western blotting analysis was performed as previously reported. Briefly, cells were lysed in immunoprecipitation lysis buffer (Beyotime Biotechnology, Shanghai, China) with 1 mM PMSF on ice for 30 min. Protein concentrations were measured by the Pierce™ BCA Protein Assay Kit (Thermo Fisher Scientific, MA, USA). Equal amounts of protein were separated by SDS-PAGE and transferred onto 0.22 μm nitrocellulose membranes (Millipore, Cork, Ireland). The membranes were incubated with anti-SPP1 (Abcam, ab214050), anti-GAPDH (ABclonal Biotechnology, Hubei, China, A19056), anti-CD44 (Abcam, ab189524), anti-SMAD2/3 (Abcam, ab202445), anti-pSMAD2/3 (Abcam, ab254407), anti-STAT3 (Abcam, ab68153), and anti-pSTAT3 (Abcam, ab76315) overnight at 4 °C, and then incubated with IRDye 800 goat anti-rabbit antibody (LI-COR Biosciences, Lincoln, USA) for 1 h at room temperature. After washing off the unbound antibodies, the labeled bands were scanned by Odyssey® CLx Infrared Imaging System (LI-COR Biosciences, MA, USA).

### Fresh tissue dissociation into single cells

All the freshly resected surgical specimens were immediately washed with DPBS and divided into two equal parts. One part was processed to generate single-cell suspensions, while the other part was used for pathology examination and the following experiments. Tissue digestion was performed in a 15 mL tube containing 5 mL pre-warmed DMEM (Gibco) supplemented with enzymes in Tumor Dissociation Kit, human (Milenyi Biotec) following the manufacturer’s instructions. Cell suspensions were filtered using a 70 μm filter, and then dead cell and cell debris were removed using Debris Removal Solution (Milenyi Biotec). The cells were washed twice and suspended in PBS with 0.5% bovine serum albumin (BSA), waiting for library preparation and sequencing.

### Multiplex IHC

For fluorescent multiplex IHC analysis, a four-color fluorescence kit based on tyramide signal amplification (TSA) was used following the manufacturer’s protocol. In brief, slides were deparaffinized and rehydrated, and antigen was retrieved and treated with 3% H_2_O_2_ for 20 min, washed, and blocked using 1% BSA. Primary antibodies were added followed by TSA solution. After the last TSA cycle, slides were counterstained with DAPI at a dilution of 1:1000 for 10 min. The photographs of the stained sections were obtained by Leica TCS SP8 (Leica Biosystems, MA, USA).

### scRNA-seq data preprocessing

The off-machine 5′-expression sequencing data were demultiplexed and aligned to the human transcriptome (build GRCh38) using the Cell Ranger v2.1.1 (10× Genomics). The generated outputs for the 16 samples were pooled to obtain a combined raw expression matrix (gene counts vs cells) using the function cell ranger aggression. The unique molecular identifier (UMI) count matrix was transformed into Seurat objects using the R package Seurat (version 4.1.1). Qualified cells with detected gene numbers between 200 and 6000, UMI counts between 1000 and 50,000, and a percentage of mitochondrial genes below 10% were kept. After quality control, a dataset of 92,762 cells with 26,658 genes was obtained for downstream analysis. The raw gene expression measurements for each cell were normalized by dividing them by the total expression followed by scale factor-multiplying (×10,000) and log-transformation in (UMI-per-10,000 + 1) with the function NormalizeData in the Seurat toolkit.

To eliminate batch effects, we used a harmony algorithm to integrate samples based on patient samples^47^. Briefly, we split the combined Seurat object into a list of Seurat objects with each dataset as an element by running the command of SplitObject. Each Seurat dataset within the list was normalized for the identification of variable genes by running NormalizeData and FindVariableFeatures (SeuratObject, selection. method = “vst,” features = 2000). Next, RunHarmony was performed, which returned a Seurat object holding integrated batch effect corrected expression matrix. A “harmony” assay with the integrated expression matrix was contained in the Seurat object. The original uncorrected values were stored in the object in the “RNA” assay, ready to be switched back and forth. The new integrated expression matrix was used for downstream analysis. First, we scaled the integrated data for PCA and tSNE visualization. The cells were then clustered by cell type, rather than by batch effects. Major cell types (T cells, NK cells. myeloid cells, ECs, hepatocytes, cycling cells, hepatic stellate cells, plasma cells, and B cells) were identified by mapping canonical marker genes in the two-dimensional UMAP map.

We obtained the processed CRC public scRNA-seq dataset from Qi et al.^27^ by downloading Supplementary Data S3 and S4 from the published article.

### Analysis of functional cellular subsets within the major cell types

Functional sub-clusters within each of the major cell types were partitioned and analyzed. We performed PCA using the variably expressed genes for each major cell type object under the “integrated” assay mode. Significant principal components, selected by looking at the elbow position on standard deviation graphs of PCAs, were used for subsequent clustering and UMAP visualization. Seurat function FindClusters was utilized with a suitable resolution to identify sub-clusters within each major cell type. Cells with an expression of double-lineage genes, including CD3^+^CD19^+^ cells, and CD68^+^CD19^+^ cells, were excluded to eliminate potential doublet capture bias. DEGs for each of the sub-clusters were identified using the FindAllMarkers function under RNA assay mode. The test method used for FindAllMarkers was the Wilcoxon rank sum test. We defined each cell sub-cluster based on the expression of canonical markers. Any transcriptomic comparisons, e.g., between APHC and ANHC, between tumor and ANT groups, were performed using the function FindMarkers (object = obj, ident.1 = ident.1, ident.2 = ident.2, verbose = FALSE) under RNA assay mode Pseudo-time trajectory analysis.

Single-cell trajectory analysis was performed with the Monocle2 package (v2.8.0). The starting subject CellDataSet class was created with normalized expression cells × gene matrix. The variable genes are subsequently used to define cell progress. DDRTree incorporated in the reduceDimension function was applied for data dimension reduction. Cells were then ordered in pseudo time with the order cell function. The trajectory was visualized in two-dimensional space by running the plot_cell_trajectory function.

### TF activity analysis

To measure TF activity, pySCENIC was applied to deduce gene regulatory networks across cell clusters. Briefly, TFs and their target genes, together defining a regulon, are derived using gene inference met that solely relies on correlations between the expression of genes across cells in the arboreta package. These regulons are refined by pruning targets that do not have enrichment for a corresponding motif of the TF effectively separating direct from indirect targets based on the presence of *cis*-regulatory footprints. Finally, the original cells are differentiated and clustered on the activity of these discovered regulons.

### Mass CyTOF and data processing

The pre-conjugated antibodies encompassing 34 markers were purchased from the supplier (Cat# 201321, 201307, and 201305 (Fludigm, USA)). Tumor-infiltrating lymphocytes (3 × 10^6^) were isolated from freshly resected HCC samples and stained for viability with 5 μM Cisplatin for 2 min, and then incubated with surface markers for 30 min at room temperature. Cells were fixed and analyzed with a Helios mass cytometer (Fludigm, USA) finally. For each sample, 500,000–1,000,000 cell events were collected. Exported files (.fcs) were uploaded into Cytobank (https://community.cytobank.org), total T cells were manually gated, and events of interest were exported as.fcs files. The high-dimensional raw data were processed with dimensional reduction before further analysis. A random sampling from each.fcs file was performed using a cytofWorkflow package on R software.

### Statistical analysis

Unpaired *t*-test and Wilcoxon rank-sum tests were applied for continuous variables in GraphPad Prism9 software and a *P*-value < 0.05 was considered statistically significant.

## Supplementary information

Supplementary information

## Data Availability

The raw scRNA-seq data reported in this study have been deposited in the Genome Sequence Archive in the National Genomics Data Center, China National Center for Bioinformation/Beijing Institute of Genomics, Chinese Academy of Sciences, and are publicly accessible at https://ngdc.cncb.ac.cn/gsa (Bioproject accession number: PRJCA016727; GSA-Human: HRA004584).
