# Supplementary material for: Multi-dimensional single-cell characterization revealed suppressive immune microenvironment in AFP-positive hepatocellular carcinoma
Source: Cell Discov. 2023 Jun 19;9:60. doi: 10.1038/s41421-023-00563-x (PMC10279759; doi:10.1038/s41421-023-00563-x)
Supplement: Supplementary file 1 — Supplementary information [file 41421_2023_563_MOESM1_ESM.pdf]

Supplementary Information

Multi-dimensional Single-cell Characterization Revealed Suppressive Immune Microenvironment in AFP-positive Hepatocellular Carcinoma

Supplementary Fig. S1 -- **P2-3**

Supplementary Fig. S2 -- **P4-5**

Supplementary Fig. S3 -- **P6-8**

Supplementary Fig. S4 -- **P9-10**

Supplementary Fig. S5 --- **P11-13**

Supplementary Fig. S6 --- **P14-15**

Supplementary Fig. S7 -- **P16-17**

Supplementary Table. S1 -- **P18-19**

Supplementary Table. S2 -- **P20-21**

Supplementary Table. S3 -- **P22**

Supplementary Table. S4 -- **P23**

Supplementary Table. S5 -- **P24**

Supplementary Figures

Supplementary Fig. S1

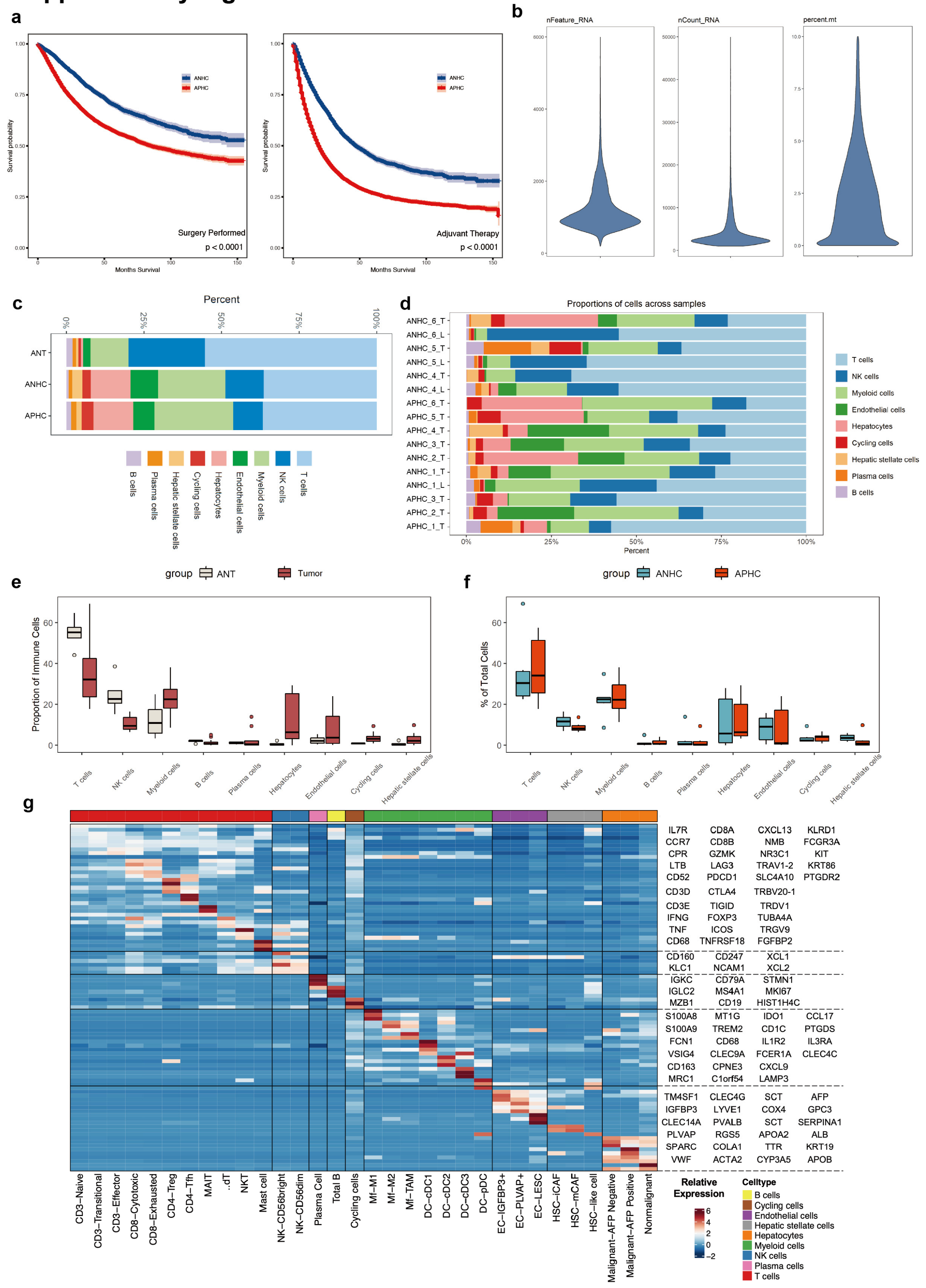

Supplementary Fig. S1 Subgroup analysis of SEER cohort and details in the scRNA-seq cohort.

a Overall survival of patients dispelled as Kaplan-Meier curves according to different subgroups: Surgery performed (left) and adjuvant performed (right).

b Violin plots showing the number of genes (nFeature_RNA), number of reads (nCount_RNA), and percent of mitochondrial-derived transcripts (percent. mt) per single cell after strict quality control.

c Bar plots showing 9 major cell lineage distributions within different groups. (ANT, adjacent normal tissue; APHC, AFP-Positive HCC; ANHC, AFP-Negative HCC)v

d Representation of each sample within each major cell type.

e Boxplot illustrating the fraction of 9 major cell types in tumors and ANT.

f Boxplot illustrating the fraction of 9 major cell types in ANHC and APHC.

g Heatmap displaying average expressions of marker genes for 38 cell subsets.

Supplementary Fig. S2

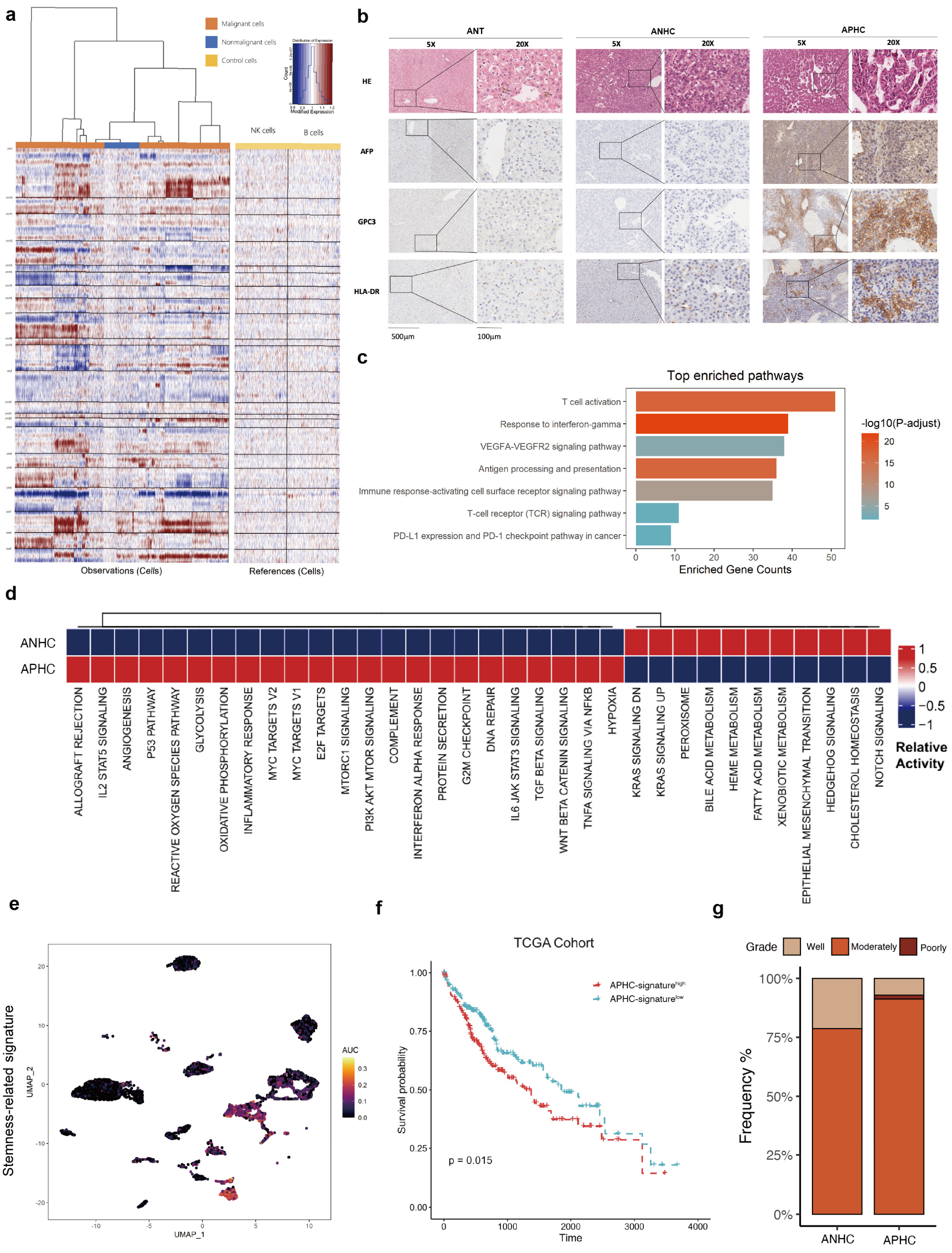

Supplementary Fig. S2 Characteristics of malignant cells in ANT, ANHC, and APHC.

a Heatmap showing CNVs of single cells inferred from scRNA-seq data.

b The IHC staining of HLA-DR in ANT, ANHC, and APHC.

c Enriched pathways of HLA-DRA and CD74 double-positive malignant cells.

d Heatmap showing the difference in pathway activities scored by GSVA per cell between groups.

e Individual cell AUC score overlay for selected stemness-related genes.

f Kaplan-Meier plots of overall survival in patients from TCGA dataset defined by APHC-signature.

g The distribution of HCC with different differentiation between groups based on the EHBH cohort.

Supplementary Fig. S3-1

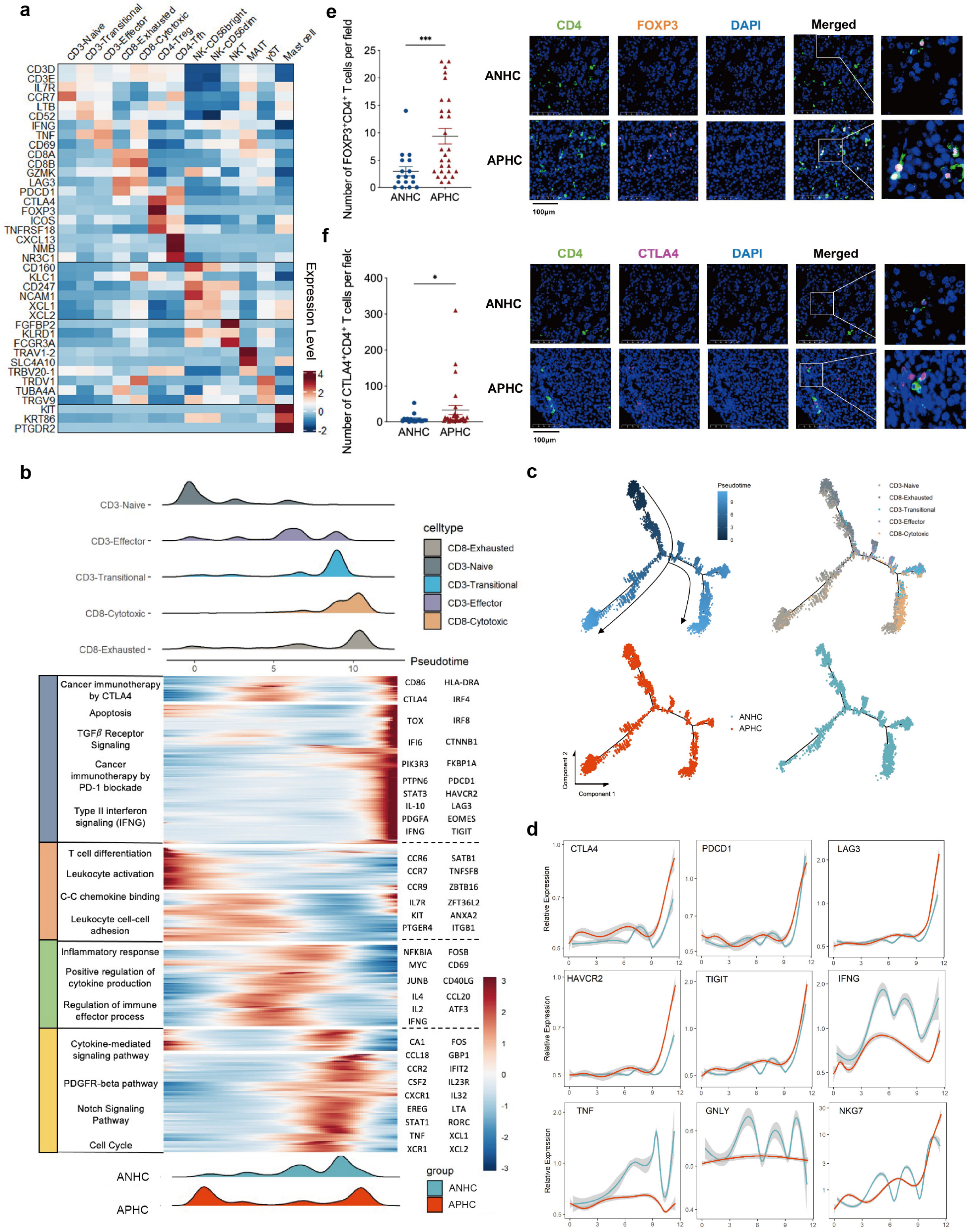

Supplementary Fig. S3-2

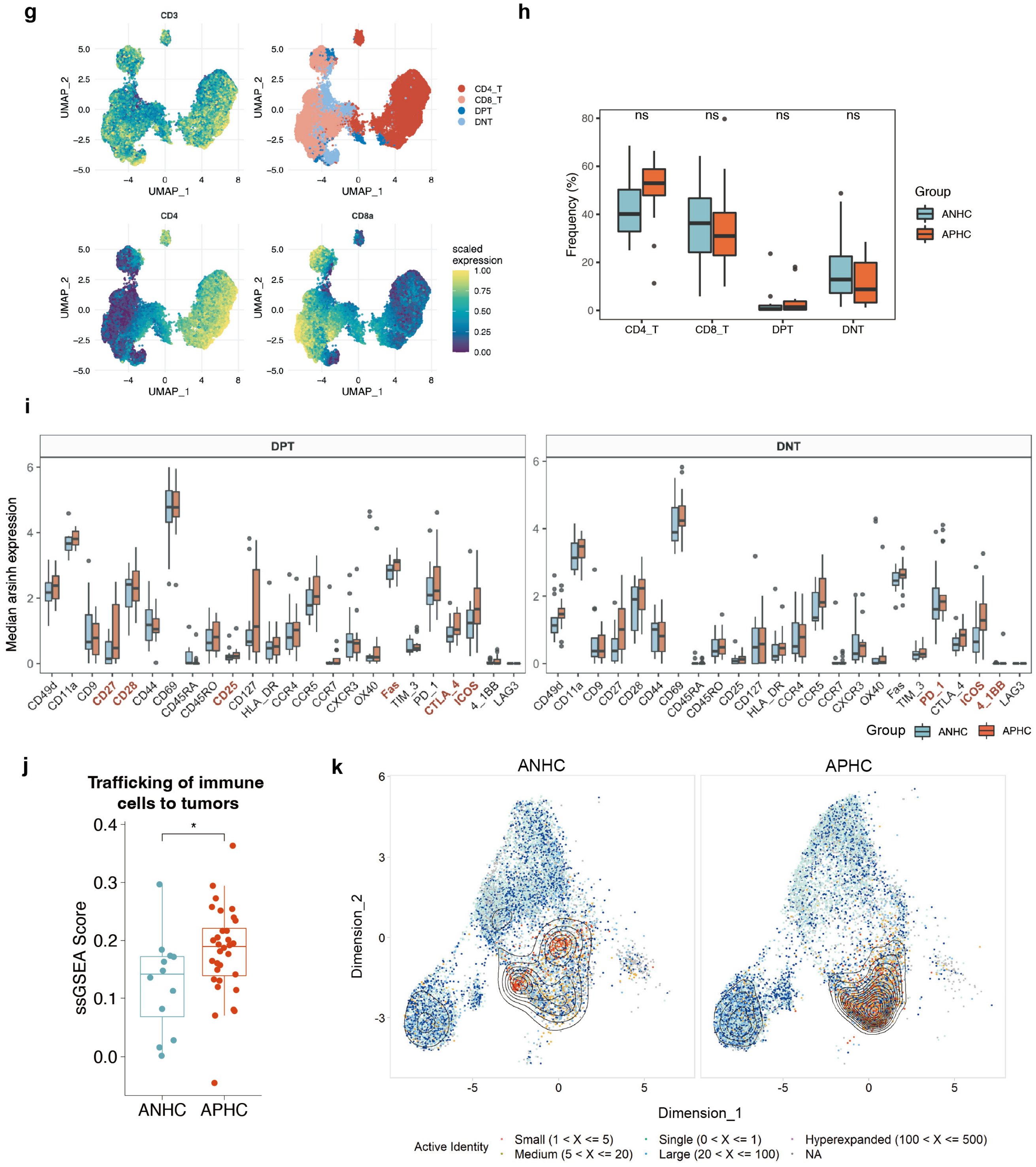

Supplementary Fig. S3 Analysis of T cell transition and expansion in ANHC and APHC.

a Average expression of T/NK cell-specific markers across different cell counterparts.

b Heatmap showing the dynamic changes in gene expression of total $CD8^+$ T cells along the pseudotime. The distribution of $CD8^+$ T subsets (upper panel) and total $CD8^+$ T across groups (lower panel) along with pseudotime.

c Two-dimensional graph of the pseudotime-ordered $CD8^+$ T cells.

d Two-dimensional plots showing the expression for genes related to T cell function, in ANHC and APHC, along with the pseudotime.

e The proportions of Tregs between each group and the corresponding representative images.

f The proportions of $CD4^+CTLA4^+$T cells between each group and the corresponding representative images.

g UMAP plots showing the identified 4 T-cell subsets in CyTOF datasets. (DPT: double-positive T cells; DNT: double-negative T cells)

h The distribution of 4 T-cell subsets between groups based on the CyTOF cohort.

i Relative expression levels of each functional marker of DPT ($CD4^+$ $CD8^+$ T cell) and DNT ($CD4^-$ $CD8^-$ T cell) across recruited CyTOF cohort.

j Boxplots showing the level of trafficking signature scored by ssGSEA within the EHBH bulk-RNAseq cohort.

k Contour density diagram indicating the gradient of the expanded T cell in ANHC and APHC.

Supplementary Fig. S4

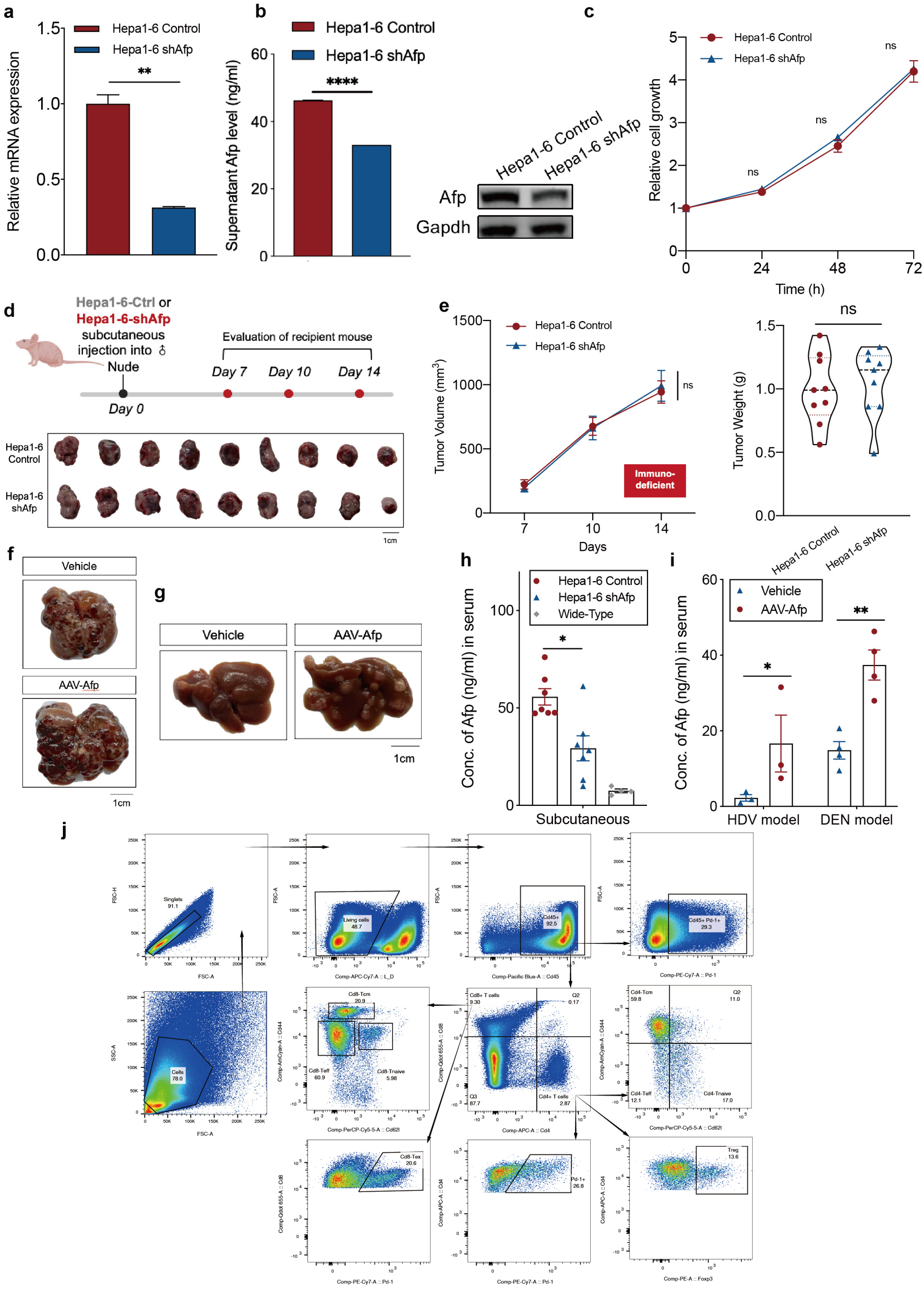

Supplementary Fig. S4 Afp was involved in modulating the immune microenvironment of HCC.

a Afp mRNA levels in Hepa1-6 cells with Afp knockdown.

b Afp protein levels in Hepa1-6 cells with Afp knockdown (left: secreting; right: total).

c Relative cell growth curve evaluated by CCK-8 assay.

d Scheme representing the experimental procedure of the subcutaneous transplantation model in immunodeficient nude mice.

e Tumor weight and tumor growth curves in immunodeficient nude mice injected subcutaneously with Control or shAfp liver cancer (Hepa1-6) for 3 weeks.

f Gross morphology of tumors in the hydrodynamic tail vein injection model.

g Gross morphology of tumors in the DEN plus CCl_4-induced HCC model.

h The level of serum Afp in the subcutaneous model.

i The level of serum Afp in HDV model and DEN model.

j Gating strategy for separation of different cellular and subcellular populations of T cells under FACS.

Supplementary Fig. S5-1

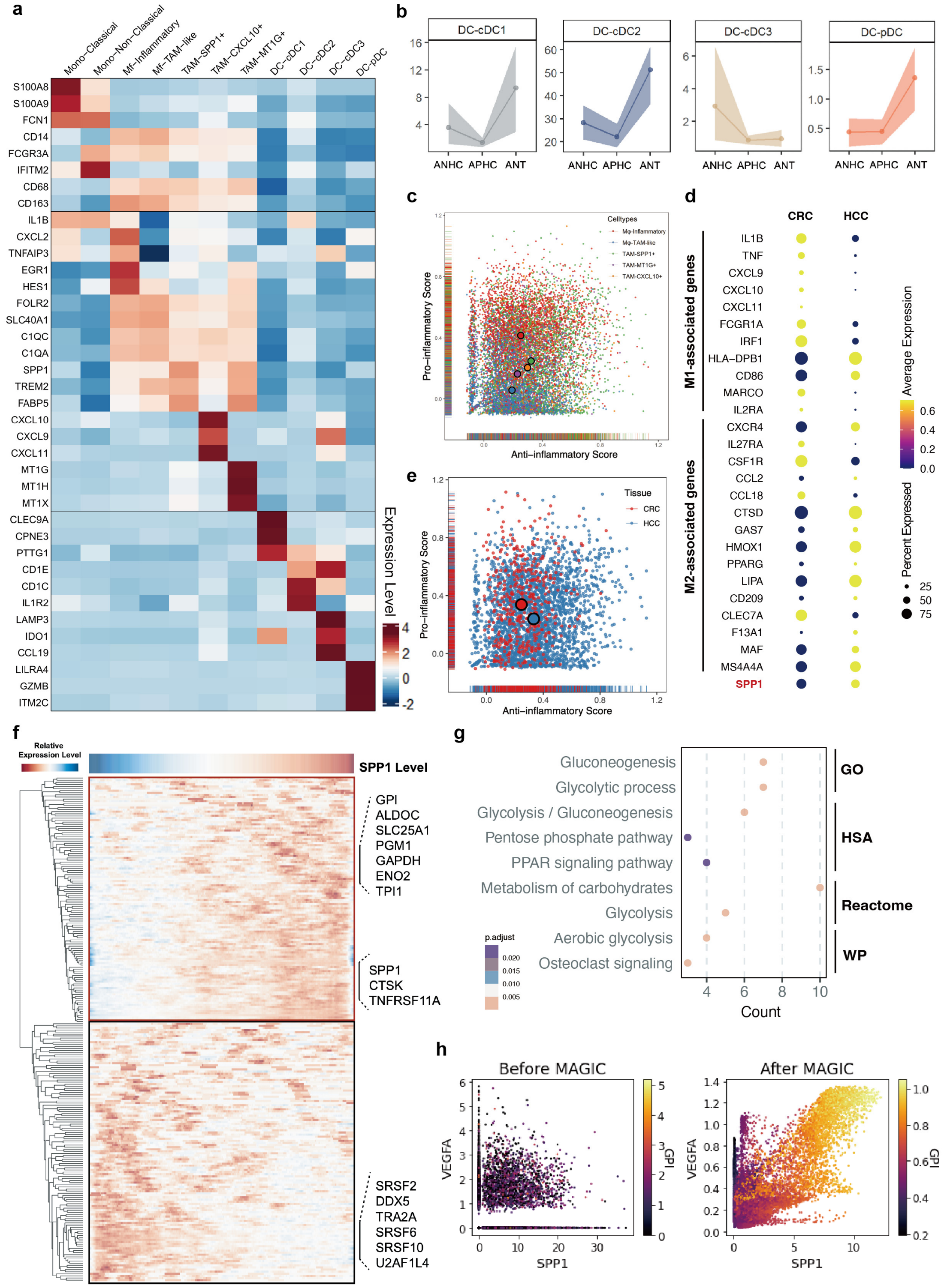

Supplementary Fig. S5-2

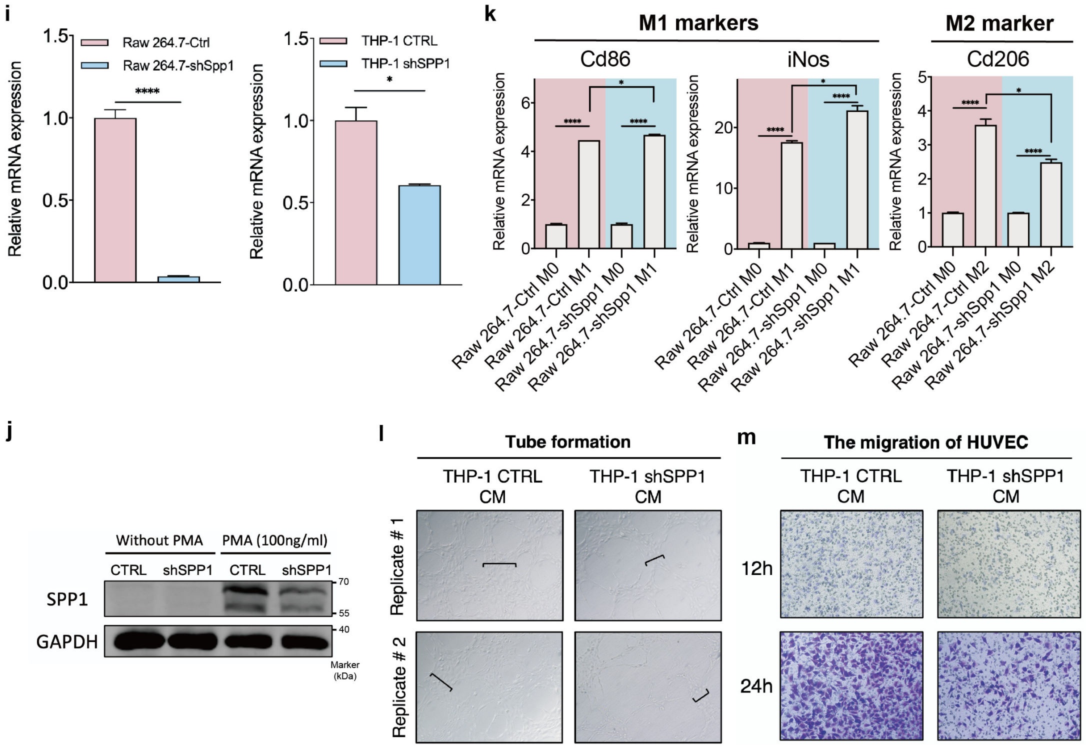

Supplementary Fig. S5 TAM-SPP1$^+$ was enriched in APHC.

a Heatmap showing average expression of myeloid cell-specific markers across different cell counterparts.

b Fractional changes for each DC subsets in ANT, ANHC, and APHC.

c Pro- and anti-inflammatory scores for each macrophage subset within HCC.

d Average expression of M1 and M2-like macrophage-related genes in SPP1$^+$TAM within HCC and CRC (see details in Methods).

e Pro- and anti-inflammatory scores for SPP1$^+$TAM within HCC and CRC.

f Scaled expression of genes with high knnDREMI conditioned on SPP1 (rows), with cells ordered by SPP1 expression (columns). The red box, the SPP^{high} module; the black box, the SPP^{low} module.

g Enriched pathways of cells in SPP^{high} module, related to Figure S5c.

h Two-dimensional scatterplot of the correlation between VEGFA, SPP1, and GPI. GPI: Glucose-6-phosphate isomerase.

i SPP1/Spp1 mRNA levels in THP-1/Raw264.7 with SPP1/Spp1 knockdown.

j SPP1/Spp1 protein levels in THP-1 with SPP1/Spp1 knockdown.

k Relative mRNA expression of M1 or M2-associated genes after induced by the corresponding molecule. (M1-like phenotype, LPS [100ng/mL, 48h]; M2-like phenotype, IL-4 [20ng/mL, 48h] & IL-10 [20ng/mL, 48h]).

l Representative images showing the tube formation of HUVEC and the brackets indicate the thickness of the vessel wall.

m Representative images showing the migration of HUVEC under different CM.

Supplementary Fig. S6

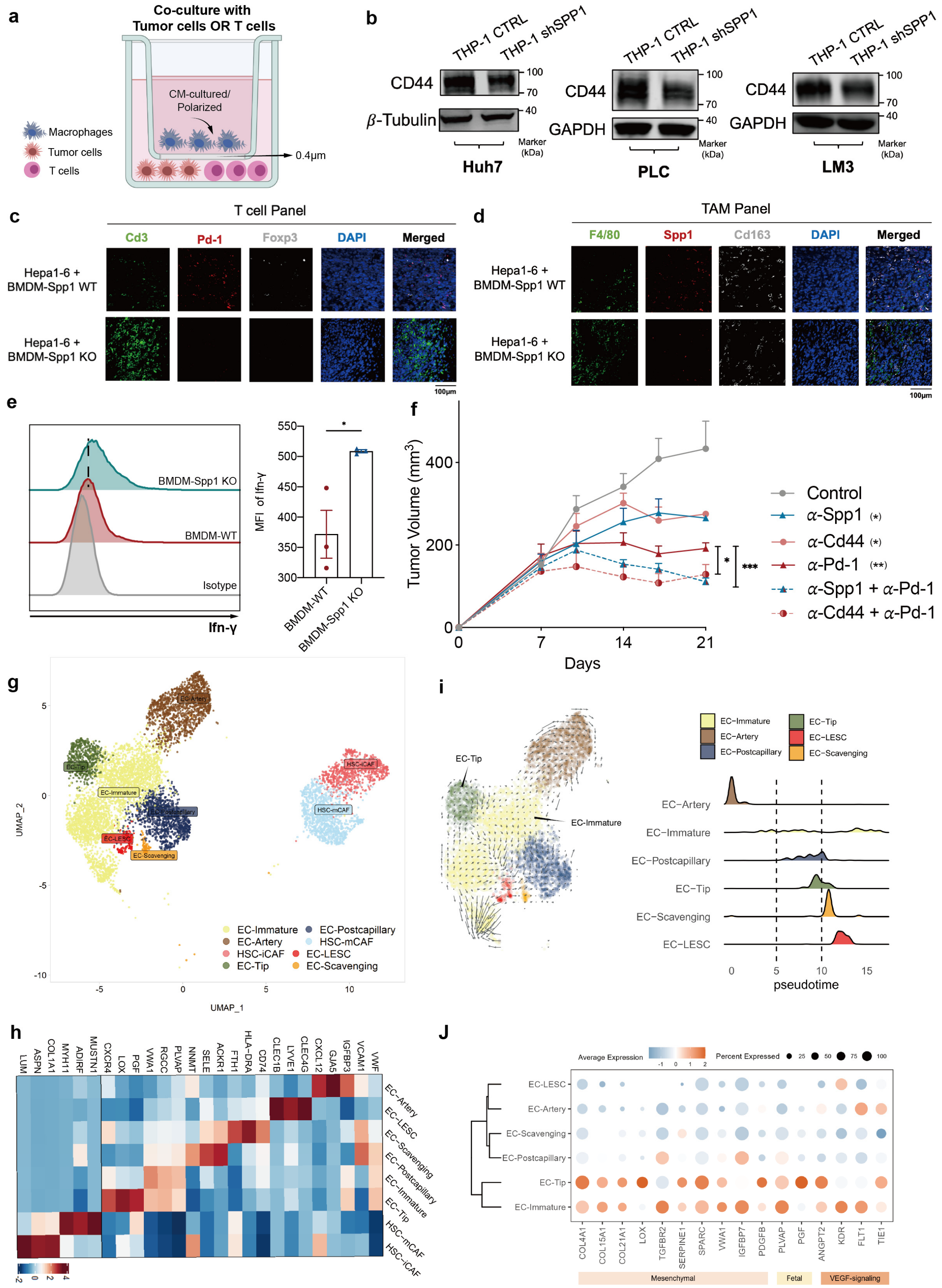

Supplementary Fig. S6 TAM-SPP1$^+$ promoted tumor progression via impairing T-cell immunity and the heterogeneity of stromal cells in the tumor microenvironment.

a Scheme representing the experimental procedure of in vitro experiments.

b The relative protein level of CD44 of HCC cell lines after co-cultured with THP-1 CTRL or THP-1 shSPP1 for 24h.

c Representative multiplex IHC staining of T cells in the subcutaneous co-transplantation model.

d Representative multiplex IHC staining of TAMs in the subcutaneous co-transplantation model.

e Mean fluorescence intensity (MFI) of Ifn-γ of Cd8$^+$ T cells co-cultured with indicated BMDM derived from wide type C57BL/6J and Spp1$^{-/-}$ C57BL/6J mice relatively. (BMDMs were pre-stimulated with tumor-derived conditioned medium for 24h)

f The growth curve of an in vivo drug administration model.

g UMAP plot showing sub-clustering of stromal cells (i.e., HSC and EC)

h Heatmap showing average expression of stromal cell-specific markers across different cell counterparts.

i RNA velocity (left) and Monocle2 analysis (right) revealed the potential transition between EC-Tip and EC- Immature.

j Dot plot showing the average expression of genes associated with matrix remodeling across different clusters.

Supplementary Fig. S7

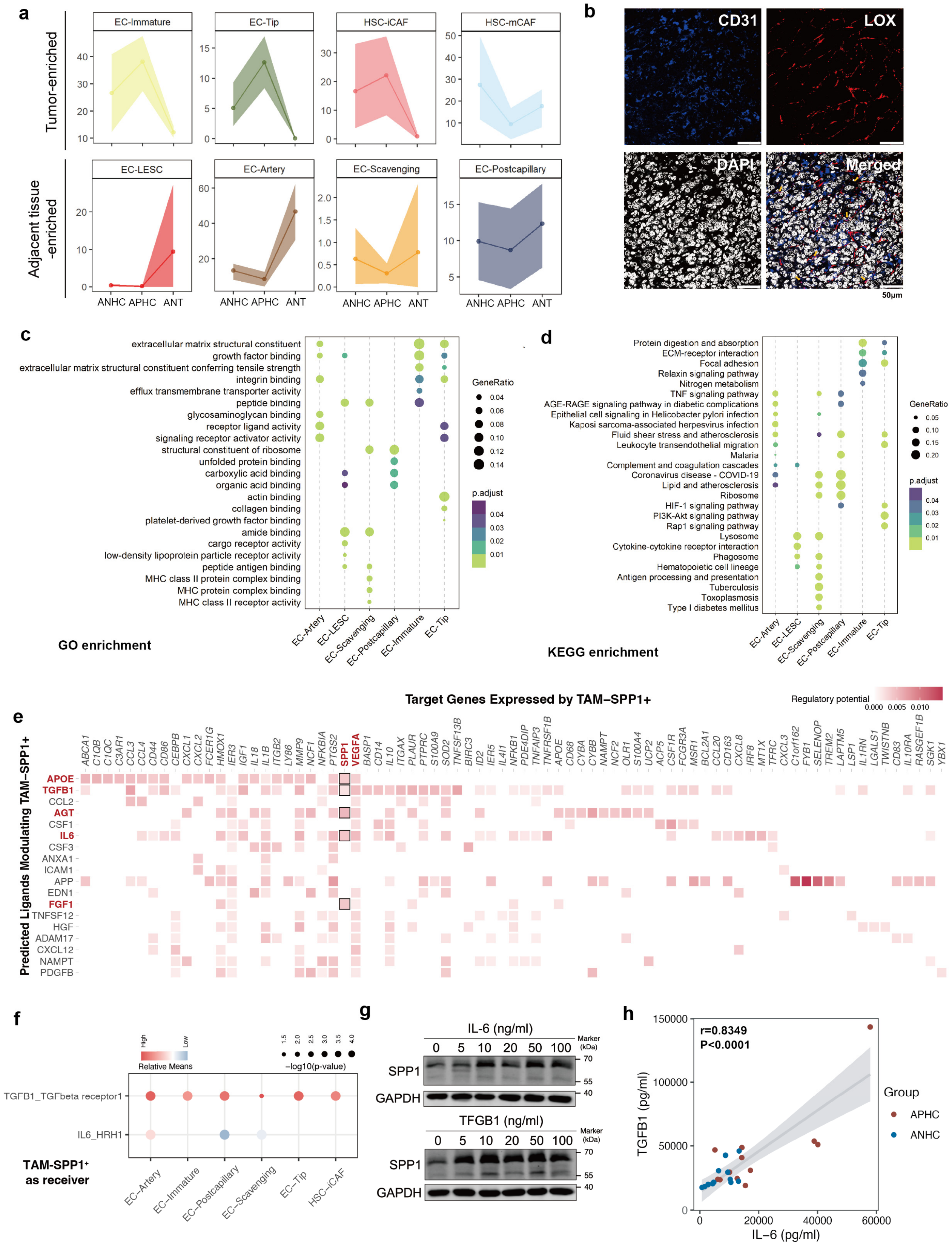

Supplementary Fig. S7 TGFB1 and IL6 signaling promoted the emergence of TAM-SPP1^{+}.

a Fractional changes for each HSC and EC subset across groups.

b The multiplex IHC staining of LOX^{+} EC confined the existence of EC-Tips in the tumor microenvironment.

c Gene ontology (GO) term enrichment analysis of each EC subset.

d KEGG enrichment analysis of each EC subset.

e Heatmap showing the potential ligands from stromal cells and the corresponding targeted gene in TAM-SPP1^{+}.

f Ligand-receptor pairs involved in TAM-SPP1^{+} activation was predicted by *CellPhoneDB.*

g TGFB1 (left) or IL6 (right) did not induce SPP1 expression in PMA-stimulated THP-1 in a dose-dependent manner. Protein expression by western blot analysis at 16h after treatment.

h Correlation of TGFB1 and IL6 from the data obtained from ELISA.

Supplementary Table. S1 Baseline characteristics of patients with HCC from SEER database

Variables	Overall (n = 55422)	ANHC (n = 13990)	APHC (n = 41432)	P-value
Age, y				<0.001*
< 60	21469 (38.7%)	4771 (34.1%)	16698 (40.3%)	
≥ 60	33953 (61.3%)	9219 (65.9%)	24734 (59.7%)	
Age at diagnosis, y	62.0 [56.0;71.0]	64.0 [57.0;72.0]	62.0 [56.0;70.0]	<0.001*
Gender				0.003*
Female	12630 (22.8%)	3059 (21.9%)	9571 (23.1%)	
Male	42792 (77.2%)	10931 (78.1%)	31861 (76.9%)	
Grade				<0.001*
Well differentiated; Grade I	5955 (31.0%)	2488 (41.9%)	3467 (26.1%)	
Moderately differentiated; Grade II	8676 (45.1%)	2644 (44.5%)	6032 (45.4%)	
Poorly differentiated; Grade III	4233 (22.0%)	747 (12.6%)	3486 (26.2%)	
Undifferentiated; anaplastic; Grade IV	361 (1.88%)	66 (1.11%)	295 (2.22%)	
Fibrosis Score[a]				<0.001*
F0: Fibrosis 0-4; None to moderate fibrosis	2932 (18.4%)	979 (22.0%)	1953 (17.0%)	
F1: Fibrosis 5-6; Severe fibrosis or cirrhosis	13013 (81.6%)	3478 (78.0%)	9535 (83.0%)	
Number of tumors				<0.001*
Multiple	8328 (15.0%)	2599 (18.6%)	5729 (13.8%)	
Single	47094 (85.0%)	11391 (81.4%)	35703 (86.2%)	
T stage[b]				<0.001*
T0	30 (0.11%)	8 (0.11%)	22 (0.11%)	
T1	12309 (44.6%)	4367 (58.0%)	7942 (39.6%)	
T2	6627 (24.0%)	1840 (24.4%)	4787 (23.9%)	
T3a	4103 (14.9%)	737 (9.78%)	3366 (16.8%)	
T3b	3342 (12.1%)	398 (5.28%)	2944 (14.7%)	
T3NOS	55 (0.20%)	12 (0.16%)	43 (0.21%)	
T4	1108 (4.02%)	171 (2.27%)	937 (4.68%)	
N stage[b]				<0.001*
N0	25656 (92.0%)	7234 (95.3%)	18422 (90.7%)	
N1	2243 (8.04%)	353 (4.65%)	1890 (9.30%)	
M stage[b]				<0.001*
M0	26065 (86.4%)	7338 (92.0%)	18727 (84.4%)	
M1	4092 (13.6%)	634 (7.95%)	3458 (15.6%)	
Extension				<0.001*
Confined to liver, without intrahepatic vascular invasion	25872 (55.8%)	7963 (68.0%)	17909 (51.7%)	
Confined to liver, with intrahepatic vascular invasion	12952 (28.0%)	2721 (23.2%)	10231 (29.6%)	
Major vascular invasion	5747 (12.4%)	701 (5.98%)	5046 (14.6%)	
Extrahepatic metastases	1757 (3.79%)	328 (2.80%)	1429 (4.13%)	

Adjuvant Therapy				0.001*
Chemotherapy	20893 (82.8%)	5280 (83.8%)	15613 (82.4%)	
Radiation	2454 (9.72%)	612 (9.72%)	1842 (9.73%)	
Both	1890 (7.49%)	405 (6.43%)	1485 (7.84%)	
Surgery				<0.001*
Surgery performed	13650 (24.7%)	4878 (35.0%)	8772 (21.2%)	
Not performed	41599 (75.3%)	9062 (65.0%)	32537 (78.8%)	
Vital status[c]				0.000*
Alive	14710 (26.5%)	5633 (40.3%)	9077 (21.9%)	
Deceased	40712 (73.5%)	8357 (59.7%)	32355 (78.1%)	

Abbreviation: HCC, Hepatocellular carcinoma; AFP, Alpha-fetoprotein; NOS, Not otherwise specified

[a] Fibrosis Score is also called Ishak Score

[b] AJCC Cancer Stage 7th Edition

[c] Not HCC-specific survival

* P- value was significant for 0.05

Supplementary Table. S2 Baseline characteristics of patients with HCC in EHBH cohort

	Overall (n = 297)	ANHC (n = 85)	APHC (n = 212)	P-value
Age, y	47.0 [41.0;56.0]	51.0 [43.0;58.0]	46.0 [40.0;54.0]	0.014*
Gender				0.135
Male	261 (87.9%)	79 (92.9%)	182 (85.8%)	
Female	36 (12.1%)	6 (7.06%)	30 (14.2%)	
Grade				0.022*
Grade II	29 (9.93%)	14 (16.7%)	15 (7.21%)	
Grade III	261 (89.4%)	69 (82.1%)	192 (92.3%)	
Grade IV	2 (0.68%)	1 (1.19%)	1 (0.48%)	
Number of tumors				0.107
Multiple	33 (11.1%)	5 (5.88%)	28 (13.2%)	
Single	264 (88.9%)	80 (94.1%)	184 (86.8%)	
Diameter, cm	6.00 [4.00;9.00]	4.50 [3.50;6.00]	7.00 [4.00;10.0]	<0.001*
Transverse diameter, cm	4.50 [3.00;7.00]	3.50 [3.00;5.00]	5.00 [3.00;7.50]	<0.001*
Cirrhosis				0.100
Negative	83 (27.9%)	30 (35.3%)	53 (25.0%)	
Positive	214 (72.1%)	55 (64.7%)	159 (75.0%)	
Pathology				<0.001*
Thick trabecular pattern	235 (79.1%)	54 (63.5%)	181 (85.4%)	
Pseudoglandular pattern	8 (2.69%)	7 (8.24%)	1 (0.47%)	
Clear cell pattern	10 (3.37%)	5 (5.88%)	5 (2.36%)	
Clustered pattern	5 (1.68%)	2 (2.35%)	3 (1.42%)	
Thin trabecular pattern	36 (12.1%)	17 (20.0%)	19 (8.96%)	
Solid pattern	3 (1.01%)	0 (0.00%)	3 (1.42%)	
Hemorrhagic necrosis				0.225

	Overall (n = 297)	ANHC (n = 85)	APHC (n = 212)	P-value
Negative	88 (29.6%)	30 (35.3%)	58 (27.4%)	
Positive	209 (70.4%)	55 (64.7%)	154 (72.6%)	
Satellite				0.913
Negative	154 (51.9%)	45 (52.9%)	109 (51.4%)	
Positive	143 (48.1%)	40 (47.1%)	103 (48.6%)	
MVI				0.508
Negative	133 (44.8%)	35 (41.2%)	98 (46.2%)	
Positive	164 (55.2%)	50 (58.8%)	114 (53.8%)	
PVE				0.001*
Negative	138 (46.6%)	54 (63.5%)	84 (39.8%)	
Thrombus in gross	35 (11.8%)	6 (7.06%)	29 (13.7%)	
Microscopic thrombus	123 (41.6%)	25 (29.4%)	98 (46.4%)	
Capsule				0.043
Complete	91 (30.6%)	35 (41.2%)	56 (26.4%)	
Incomplete	78 (26.3%)	18 (21.2%)	60 (28.3%)	
No capsule	128 (43.1%)	32 (37.6%)	96 (45.3%)	
Extrahepatic metastases				0.646
Negative	256 (86.2%)	75 (88.2%)	181 (85.4%)	
Positive	41 (13.8%)	10 (11.8%)	31 (14.6%)	

Abbreviation: HCC, Hepatocellular carcinoma; AFP, Alpha-fetoprotein; PVE, Portal Vein Embolization; MVI, Microvascular invasion (* P- value was significant for 0.05)

Supplementary Table. S3 Summary of patient cohorts for scRNA-seq analysis

Case No.	Cell viability of tumors	Cell viability of adjacent normal tissue	Gender	Age	Tumor size (mm)	AFP level (<20 μg/L)	HBV-DNA (<50 IU/ml)	Edmondson stage	MVI Stage	Cirrhosis[a]
APHC_1_T	76.80%	-	M	46	117×79	> 1210	2.07E+06	III	M1	0
APHC_2_T	77%	-	M	62	98×79	> 1210	6.52E+06	III	M1	1
APHC_3_T	88%	-	M	55	104×82	> 1210	4.39E+02	III	M1	1
ANHC_1_T	70.30%	82.70%	M	47	40×31	1.8	2.72E+02	III	M0	0
ANHC_2_T	66%	-	M	55	40×36	8.9	< 50	III	M0	0
ANHC_3_T	68.70%	-	M	54	101×88	1.5	< 50	III	M0	1
APHC_4_T	75.30%	-	F	23	87×73	> 1210	1.76E+04	III	M0	0
APHC_5_T	80%	-	F	65	82×64	> 1210	< 50	III-IV	M1	0
APHC_6_T	75.30%	-	F	56	135×131	> 1210	< 50	III	M1	1
ANHC_4_T	72%	78%	F	66	111×106	5.4	< 50	III	M0	1
ANHC_5_T	78.50%	91%	F	69	27×24	1.7	< 50	III	M1	1
ANHC_6_T	85.30%	86.80%	F	51	24×20	1.6	< 50	III	M0	0

Supplementary Table. S4 Primers for Real-time quantitative PCR

**Primer name**	**Property**	**Sequence**
SPP1	Forward primer	5'-CTCCATTGACTCGAACGACTC-3'
	Reverse primer	5-CAGGTCTGCGAAACTTCTTAGAT-3'
CD44	Forward primer	5'-CTGCCGCTTTGCAGGTGTA-3'
	Reverse primer	5'-CATTGTGGGCAAGGTGCTATT-3'
Spp1	Forward primer	5'-AGCAAGAAACTCTTCCAAGCAA-3'
	Reverse primer	5'-GTGAGATTCGTCAGATTCATCCG-3'
Cd86	Forward primer	5'-TGTTTCCGTGGAGACGCAAG-3'
	Reverse primer	5'-TTGAGCCTTTGTAAATGGGCA-3'
iNOS	Forward primer	5'-GTTCTCAGCCCAACAATACAAGA-3'
	Reverse primer	5'-GTGGACGGGTCGATGTCAC-3'
Cd206	Forward primer	5'-CTCTGTTCAGCTATTGGACGC-3'
	Reverse primer	5'-CGGAATTTCTGGGATTCAGCTTC-3'

Supplementary Table. S5 Metal-conjugated antibodies used in CyTOF analysis

Isotype	Antigen	Clone	Source
Pr141	CD49d	9F10	Fluidigm
Nd142	CD11a	HI111	Fluidigm
Nd143	CD5	UCHT2	Fluidigm
Nd144	CCR5	NP-6G4	Fluidigm
Nd145	CD4	RPA-T4	Fluidigm
Nd146	CD8a	RPA-T8	Fluidigm
Sm147	CD7	CD7-6B7	Fluidigm
Sm148	CD16	3G8	Fluidigm
Sm149	CD25	2A3	Fluidigm
Nd150	OX40	ACT35	Fluidigm
Eu151	CD2	TS1/8	Fluidigm
Sm152	Fas	DX2	Fluidigm
Eu153	TIM-3	F38-2E2	Fluidigm
Sm154	CD45	HI30	Fluidigm
Gd155	PD-1	EH12.2H7	Fluidigm
Gd156	CXCR3	G025H7	Fluidigm
Gd158	CCR4	L291H4	Fluidigm
Tb159	CCR7	G043H7	Fluidigm
Gd160	CD28	CD28.2	Fluidigm
Dy161	CTLA-4	14D3	Fluidigm
Dy162	CD69	FN50	Fluidigm
Dy164	CD161	HP-3G10	Fluidigm
Ho165	CD45RO	UCHL1	Fluidigm
Er166	CD44	BJ18	Fluidigm
Er167	CD27	O323	Fluidigm
Er168	ICOS	C398.4A	Fluidigm
Tm169	CD45RA	HI100	Fluidigm
Er170	CD3	UCHT1	Fluidigm
Yb171	CD9	SN4 C3-3A2	Fluidigm
Yb172	CD57	HCD57	Fluidigm
Yb173	4-1BB	4B4-1	Fluidigm
Yb174	HLA-DR	L243	Fluidigm
Lu175	LAG3	11C3C65	Fluidigm
Yb176	CD127	A019D5	Fluidigm
